# Smart Ring in Clinical Medicine: A Systematic Review

**DOI:** 10.3390/biomimetics10120819

**Published:** 2025-12-05

**Authors:** Eun Jeong Gong, Chang Seok Bang, Jae Jun Lee, Gwang Ho Baik

**Affiliations:** 1 Department of Internal Medicine, Hallym University College of Medicine, Chuncheon 24253, Republic of Korea; gong-eun@hanmail.net (E.J.G.);; 2 Institute for Liver and Digestive Diseases, Hallym University, Chuncheon 24253, Republic of Korea; 3 Institute of New Frontier Research, Hallym University College of Medicine, Chuncheon 24253, Republic of Korea; 4 Department of Anesthesiology and Pain Medicine, Hallym University College of Medicine, Chuncheon 24253, Republic of Korea

**Keywords:** smart ring, wearable devices, digital health, remote monitoring, clinical medicine

## Abstract

Background: Smart rings enable continuous physiological monitoring through finger-worn sensors. Despite growing consumer adoption, their clinical utility beyond sleep tracking remains unclear. Objectives: To systematically review evidence for smart ring applications in clinical medicine, assess measurement accuracy, and evaluate clinical outcomes. Methods: We searched PubMed/MEDLINE, Embase, Cochrane Library, and Web of Science through 31 July 2025. Two reviewers independently screened studies and extracted data. Risk of bias was assessed using ROBINS-I and RoB 2.0. Results: From 862 citations, 107 studies met inclusion criteria including approximately 100,000 participants. Studies were equally distributed between sleep (47.7%) and non-sleep applications (52.3%). Smart rings demonstrated high accuracy: heart rate r^2^ = 0.996, heart rate variability r^2^ = 0.980, and sleep detection 93–96% sensitivity. Predictive capabilities included COVID-19 detection 2.75 days pre-symptom (82% sensitivity), inflammatory bowel disease flare prediction 7 weeks early (72% accuracy), and bipolar episode detection 3–7 days early (79% sensitivity). However, 65% of studies had moderate-to-high bias risk. Limitations included small samples, proprietary algorithms (89%), poor diversity reporting (35%), and declining adherence (80% at 3 months to 43% at 12 months). Conclusion: Smart rings have evolved into clinical tools capable of early disease detection. However, algorithmic opacity, population homogeneity, and adherence challenges require attention before widespread implementation.

## 1. Introduction

Advances in digital health technologies have enabled continuous monitoring of behavioral and physiological signals through wearable devices [1]. These devices collect real-time data on individuals’ daily activity, heart rate (HR), sleep, and other health metrics, providing a more comprehensive view of patient health than traditional sporadic measurements [1]. The global wearable device market is now a multi-billion-dollar industry [2,3,4]. Wearables come in various forms—smartwatches, activity bands, patches, ear buds, clothing-embedded sensors, and rings—that incorporate sensors to measure step counts, physical activity intensity, movement patterns, HR, and rhythm, blood pressure, oxygen saturation, sleep, and body temperature, generating rich longitudinal datasets that may reveal subtle physiological changes preceding clinical manifestations [1,5]. Devices may be classified as consumer-grade, research-grade or medical-grade [6].

Recent comprehensive reviews demonstrate that wearables have progressed from simple activity tracking to sophisticated cardiovascular monitoring, with demonstrated applications in arrhythmia detection, heart failure management, and post-surgical recovery monitoring [1,2]. The global market growth reflects both consumer adoption and increasing clinical integration, with usage among individuals at cardiovascular risk reaching 11% in the United States by 2020 [4].

Among wearables, smart rings are emerging as discrete, finger-worn devices that leverage the finger’s rich vascular anatomy to capture high-quality photoplethysmographic signals (PPG) [7,8]. The thin skin, consistent pressure, and absence of large muscles on the finger improve optical sensor contact and reduce motion artifacts, enabling accurate measurements of HR, respiratory rate, blood oxygen saturation, and sleep cycles [7,9,10]. Finger-worn devices offer high signal clarity and are lightweight and comfortable for 24/7 monitoring [10,11]. Finger-based PPG sensors can achieve a high signal-to-noise ratio when properly placed [2,7,12]. Despite these advantages, the market of finger-worn wearables is small compared with wrist- or chest-mounted devices [9].

A recent meta-analysis of smart ring applications documented measurement accuracy comparable to medical-grade devices for heart rate and sleep parameters, though noting substantial heterogeneity in validation methodologies across studies [9]. Clinical applications have expanded beyond wellness tracking, with demonstrated utility in pregnancy monitoring where continuous physiological data successfully distinguished pregnancy outcomes and predicted labor onset [13].

The design and application of smart rings exemplify key principles of biomimetics—learning from and mimicking nature’s time-tested strategies to solve complex human challenges. Rather than imposing artificial monitoring systems onto the body, smart rings leverage evolved biological structures that nature has optimized over millions of years [7,8].

The strategic placement on the finger capitalizes on the digit’s unique vascular architecture. A dense capillary network lies beneath thin, glabrous skin with minimal subcutaneous fat. This provides an optimal optical window for PPG sensing with high signal-to-noise ratios [7,12]. This biomimetic approach extends beyond anatomical considerations: the continuous, unobtrusive monitoring enabled by smart rings mirrors the body’s own homeostatic mechanisms, which constantly survey and regulate physiological parameters without conscious awareness [1,10,11]. Just as biological systems employ distributed sensing networks to detect subtle physiological changes, smart rings function as external extensions of this innate monitoring capacity, translating autonomic fluctuations into clinically actionable data [1,5,8].

Furthermore, the evolution of wearable sensor technology toward miniaturized, ring-based form factors reflects a broader biomimetic trend in medical device development: the integration of technology with human biology in ways that respect and enhance natural physiological processes rather than disrupting them [1,6].

The circular geometry and continuous contact of smart rings enable stable signal acquisition that adapts to the body’s movements, much like how biological sensory systems maintain fidelity across diverse conditions [7,9,10]. This convergence of biological understanding and engineering innovation positions smart rings at the intersection of biomimetics and digital health, where device design is informed by the very physiological systems it seeks to monitor [6,7].

By embracing biomimetic principles—leveraging natural vascular pathways, mimicking continuous autonomous monitoring, and integrating seamlessly with the body’s form and function—smart rings represent a paradigm shift from episodic clinical measurements toward comprehensive, continuous health assessment that aligns with human biology [1,10,11].

Accuracy and reliability remain critical challenges [14]. Device algorithms must balance precision with comfort and battery life, and wearable data often require complex interpretation and clinical oversight [1]. Data privacy, regulatory and cost issues, and limited capacity of healthcare staff to monitor large data streams also hinder clinical adoption [1,14].

Although smart rings have gained attention for general wellness, their clinical utility is not well characterized [9,14]. Evidence is emerging on applications in sleep monitoring, autonomic function assessment, pain prediction, infectious-disease detection and obstetric monitoring [8,9,13].

However, critical implementation challenges including algorithmic transparency, data privacy, healthcare system integration, and cost-effectiveness remain inadequately addressed in the existing literature [1,14]. These gaps underscore the need for systematic synthesis of current evidence to guide clinical adoption and identify priority areas for future investigation.

This is the first systematic review to synthesize evidence on smart rings’ clinical utility across multiple medical domains. Our objectives were to summarize the domains in which smart rings have been evaluated, assess their measurement accuracy and clinical outcomes, identify methodological limitations, and discuss implications for healthcare integration.

## 2. Methods

This systematic review followed the PRISMA and PRISMA-S reporting guidelines [15,16] and was registered in PROSPERO (CRD420251122044).

We searched PubMed/MEDLINE, Embase, Cochrane Library and Web of Science (inception to 31 July 2025) for human studies evaluating finger-worn smart rings. Search strategies incorporated keywords and indexed terms including “smart ring,” “finger-worn,” “ring-shaped wearable,” and device names (Table 1). Gray literature sources were searched to reduce publication bias.

Eligible studies were original reports using smart rings for clinical applications. We included observational studies, diagnostic accuracy studies, trials, case series/reports, and prediction models. Technical papers without human data were excluded and only full-text English-language publications were included. The search strategy for relevant articles is described in Table 1. Two reviewers (EJ Gong and CS Bang) independently screened and extracted data and disagreements were resolved by consensus or consultation with a third author (JJ Lee). Risk of bias was assessed using ROBINS-I [17] and RoB 2 [18]. Given clinical heterogeneity, we synthesized results narratively by domain.

For studies with insufficient or missing data, we contacted corresponding authors via email to request the necessary information. Authors were given two weeks to respond, with a follow-up reminder sent to non-respondents.

## 3. Results

### 3.1. Study Selection and Inclusion

**Figure 1:**
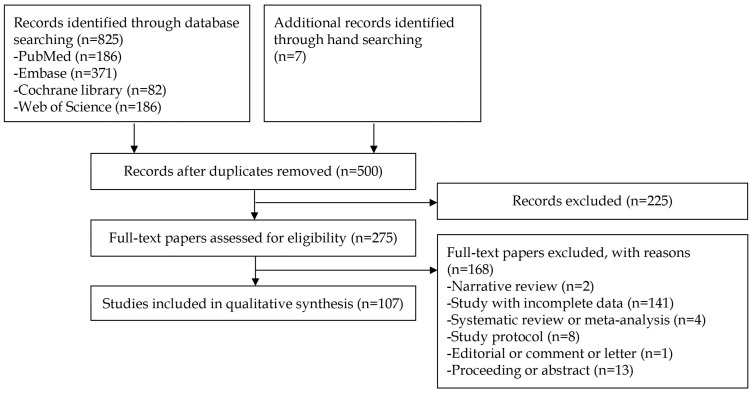
Study selection flow.

Literature search yielded 825 records from databases and 7 additional records from manual screening. After removing duplicates, 500 unique records remained. Following title/abstract screening, 275 full-text articles were assessed for eligibility. Of these, 168 were excluded. Ultimately, 107 studies met inclusion criteria (Figure 1).

### 3.2. Study Characteristics

The 107 included studies covered 85 non-randomized studies, 14 case reports/series, 8 randomized trials. Studies were published between 2019–2025, with 69.2% published in 2024–2025. Collectively enrolling approximately 100,000 participants, though skewed by large datasets (the largest 63,153 individuals). Sample sizes ranged from single cases to tens of thousands. Most studies recruited healthy volunteers, though some targeted specific patient populations. Research split almost evenly between sleep-related applications (n = 51, 47.7%) and non-sleep applications (n = 56, 52.3%). The Oura Ring featured in 72% of studies. Other devices included the Circul ring for pulse oximetry, CART-I cuffless blood pressure ring, Movano’s oxygen-sensing ring, and Moodmetric stress-tracking ring (Supplementary File).

### 3.3. Sleep Studies

#### Study Design and Populations of Sleep Studies

Among the 51 sleep studies, observational designs predominated (n = 35, 68.6%), followed by cross-sectional studies (n = 11, 21.6%), randomized studies (n = 3, 5.9%), and case reports/series (n = 2, 3.9%). Studies encompassed healthy adults (n = 8), healthcare workers (n = 3), athletes (n = 6), and clinical populations with sleep-disordered breathing, chronic pain, dementia, and post-surgical patients (Supplementary File).

### 3.4. Device Performance in Sleep Assessment

#### 3.4.1. Validation Against Polysomnography

Multiple validation studies established accuracy for sleep detection. Robbins et al. demonstrated 95% sensitivity with 83% specificity using the Oura Ring [19]. The latest Oura Ring Generation 3 showed sensitivity of 94.4–94.5% and specificity of 73.0–74.6% across 421,045 epochs [20]. However, sleep staging accuracy remained challenging at 53.18% [21]. Multi-device comparison studies revealed variable performance, with intraclass correlation coefficient of 0.77 for total sleep time but poor agreement for sleep stages [22].

#### 3.4.2. Sleep-Disordered Breathing Detection

Smart rings showed promising results for detecting sleep breathing disorders. The Circul ring demonstrated exceptional correlation (r^2^ = 0.9012) with polysomnography [23]. Zhao et al. achieved 87% sensitivity and 83% specificity for detecting apnea-hypopnea index ≥ 5 events/h with area under the curve (AUC) of 0.929 [24]. The Belun platform showed 77.6% sensitivity and 85.3% specificity for obstructive sleep apnea diagnosis [25].

### 3.5. Clinical Applications in Sleep

#### 3.5.1. Perioperative and Acute Care Settings in Sleep Studies

Sleep monitoring revealed critical clinical insights. Debbiche et al. found gynecologic surgery patients with high preoperative sleep efficiency had 3-fold lower postoperative complication rates [26]. Donahue et al. documented significant sleep architecture disruption within 72 h post-concussion [27].

#### 3.5.2. Sleep Interventions

Controlled trials evaluated sleep improvement interventions. Hausenblas et al. demonstrated magnesium-L-threonate supplementation significantly improved multiple sleep parameters [28]. Breus et al. found a pressure-releasing grid mattress improved sleep quality metrics [29]. However, Armitage et al.’s randomized trial of a sleep app showed no significant improvement [30].

### 3.6. Special Populations

Sleep patterns varied significantly across different populations and contexts. In dementia care, Ju et al. found significant correlations between sleep parameters of 11 patients and their 11 family caregivers, suggesting bidirectional sleep influences in caregiving dyads [31]. University freshmen showed markedly disrupted sleep patterns, with Soon et al. reporting bedtimes 71.76 min later than optimal and 42.6% of 638 students waking after class start times [32]. Professional athletes demonstrated context-dependent sleep variations, with Font et al. documenting moderate differences in total time in bed between home matches and travel days in elite handball players [33].

### 3.7. Large-Scale Population Sleep Studies

Willoughby evaluated 1.5 million nights of sleep data from 57,240 Oura Ring users, revealing that travel-related sleep timing disruption required more than 15 days for full recovery [34]. Viswanath et al. identified 13 distinct sleep phenotypes from 5 million nights of data in 33,152 individuals, with transition patterns differing by health status [35].

### 3.8. Non-Sleep Physiological Studies

#### Study Design and Populations of Non-Sleep Physiological Studies

Among the 56 non-sleep physiological monitoring studies, observational designs were most common (n = 35, 62.5%), followed by cross-sectional studies (n = 13, 23.2%), case reports/series (n = 6, 10.7%), and randomized controlled trials (n = 2, 3.6%). Study populations included healthy volunteers (n = 16), disease-specific cohorts including inflammatory bowel disease (IBD) (n = 309), bipolar disorder (n = 127), COVID-19 patients (n = 73), and pregnant women. Sample sizes ranged from single case reports to population-level investigations with 63,153 participants [18], demonstrating the scalability of smart ring applications from personalized medicine to public health surveillance (Supplementary File).

### 3.9. Cardiovascular and Metabolic Monitoring

#### 3.9.1. Heart Rate and Heart Rate Variability Validation

Extensive validation studies confirmed cardiac monitoring accuracy. Liang et al. demonstrated correlations exceeding 0.9 for HR variability (HRV) measurements [36]. Kinnunen et al. reported r^2^ = 0.996 for HR and r^2^ = 0.980 for HRV [37]. Age-related differences were noted, with accuracy varying between younger and older populations.

#### 3.9.2. Advanced Cardiac Applications

The CART-I ring showed exceptional performance for arrhythmia detection in 25 patients, achieving 94% overall sensitivity with 100% sensitivity for ventricular fibrillation and 90% for ventricular tachycardia detection, with intraclass correlation coefficient of 0.998 for event duration measurements [38]. For blood pressure monitoring, Kim et al. validated a cuffless smart ring system in 89 healthy adults showing minimal bias (systolic: 0.16 ± 5.90 mmHg, diastolic: −0.07 ± 4.68 mmHg) with correlation coefficients of 0.94–0.95 [39].

#### 3.9.3. Metabolic Monitoring

Novel bioimpedance technology enabled non-invasive glucose monitoring with accuracy within ±20 mg/dL for 83.6% of measurements [40]. Temperature assessments revealed connections between body temperature and depression in 20,880 individuals [41].

### 3.10. Women’s Health and Reproductive Monitoring

#### 3.10.1. Menstrual Cycle Physiology

Comprehensive menstrual cycle tracking revealed distinct physiological patterns. Alzueta et al. documented biphasic temperature and HRV across 117 women, with HR lowest during menses [42]. Maijala et al. quantified temperature differences of 0.23–0.30 °C between follicular and luteal phases using nocturnal finger skin temperature in 22 women [43]. Findings were corroborated in Olympic athletes [44].

#### 3.10.2. Ovulation and Fertility

The Oura Ring demonstrated 96.4% ovulation detection rate across 1155 ovulatory cycles from 964 women, with mean prediction error of 1.26 days compared to 3.44 days for calendar-based methods, representing a 3-fold improvement [45].

#### 3.10.3. Pregnancy Monitoring and Prediction

Continuous monitoring revealed distinct physiological trajectories. Keeler Bruce et al. tracked 120 pregnancies, identifying temperature deviations distinguishing outcomes [13]. Machine learning (ML) approaches achieved <2 days error at 8 days before labor onset [46]. Erickson et al. reported 79% accuracy within a 4.6-day prediction window [47].

### 3.11. Disease Detection and Prediction

#### 3.11.1. Infectious Disease Surveillance

Smart rings demonstrated early disease detection capabilities. For COVID-19, Mason et al. achieved 82% sensitivity with 63% specificity, detecting infections 2.75 days before testing in 63,153 participants [18]. Military applications showed similar promise with AUC 0.82 and 2.3 days lead time [48]. For influenza, Hadid et al. developed ML models achieving AUC 0.89 for predicting systemic inflammatory response [49].

#### 3.11.2. Chronic Disease Management

Continuous monitoring enabled prediction of disease exacerbations. In 309 IBD patients, Hirten et al. identified HRV patterns that differed between flare and remission states, with changes detectable 7 weeks before clinical flares [50]. For mental health conditions, Ortiz et al. demonstrated that activity variability detected depressive episodes in bipolar disorder 7.0 days earlier than sleep changes, with 79% sensitivity in 127 patients [51]. However, device limitations were noted in neurological conditions, with Reithe et al. finding poor cross-device compatibility for Parkinson’s disease monitoring in 15 patients and 16 controls [52].

### 3.12. Mental Health and Cognitive Applications

#### 3.12.1. Depression and Mood Disorders

Multiple approaches showed promise for mental health monitoring. Borelli et al. achieved F1-score of 0.744 for depression detection using multimodal passive sensing and ML in 28 undergraduates [53]. Large-scale analysis by Mason et al. revealed associations between elevated body temperature and depressive symptoms in 20,880 individuals [41]. Fudolig et al. identified two fundamental HR dynamics patterns that correlated with mental health outcomes in 599 college students [54].

#### 3.12.2. Stress and Anxiety Management

Intervention studies demonstrated measurable improvements. Balsam et al.’s mindfulness app study in 20 pregnant women showed significant reductions in stress (*p* = 0.005), anxiety (*p* = 0.01), and pregnancy-specific anxiety (*p* < 0.0001) with physiological correlates captured via Oura Ring [55]. Healthcare workers during the pandemic showed improved resilience with structured interventions monitored through wearables [56].

#### 3.12.3. Cognitive Function

Smart rings enabled objective cognitive assessment. Rovini et al. successfully differentiated 8 mild cognitive impairment patients from 10 healthy controls using reach-to-grasp motion analysis [57]. In community-dwelling older adults, Qin et al. found sleep regularity strongly correlated with executive function performance in 773 participants aged 65–80 years [58].

### 3.13. Physical Activity and Performance

#### 3.13.1. Validation Studies

Activity tracking showed variable accuracy. Kristiansson et al. reported strong correlations for step count (r = 0.93 laboratory, r ≥ 0.76 free-living) and energy expenditure validation in 32 healthy adults [59]. However, Niela-Vilen et al. found systematic overestimation in 42 adults, with Oura overestimating steps by 1416 daily and sedentary time by 17 min compared to ActiGraph accelerometer [60].

#### 3.13.2. Athletic Performance and Recovery

Smart rings provided insights into athlete physiology. Bigalke et al. demonstrated that ≥7 h sleep correlated with peak power output in 24 Division I baseball players [61]. Smith et al. revealed divergent effects of energy availability on recovery in 20 endurance athletes, with 24-h exercise-induced low energy availability extending total sleep time while diet-induced restriction reduced mean overnight heart rate [62].

### 3.14. Novel Clinical Applications

#### 3.14.1. Emergency Medicine

Smart rings enhanced emergency care delivery. Lee et al. developed a proof-of-concept device with compression depth estimation accuracy within 1.4–2.2 mm absolute error in 4 emergency medical professionals [63]. They validated its performance in the following randomized trial. Ahn et al.’s study in 20 healthy volunteers showed ring-guided cardiopulmonary resuscitation achieved 88.7% accurate rate-depth proportion versus 22.6% in controls (*p* = 0.033) [64].

#### 3.14.2. Occupational Health

Workplace implementations demonstrated feasibility and clinical utility. In US Navy shipboard personnel, Kubala et al. reported that 853 personnel used the Oura Ring for 71 ± 38% of days underway, establishing operational feasibility in challenging environments [65]. Police officers showed HRV fluctuations that predicted stress and somatization changes, as documented by de Vries et al. in 68 officers [66].

#### 3.14.3. Population Health Surveillance

Large-scale monitoring enabled syndromic surveillance. Kasl et al. demonstrated fever detection capabilities in 16,794 participants [67]. During pregnancy, continuous monitoring proved feasible with >80% adherence until late pregnancy in 15 Hispanic women, though postpartum adherence dropped to 31% [68].

#### 3.14.4. Methodological Quality

According to ROBINS-I assessment for observational studies, overall risk of bias was low in 16 studies (15.0%), moderate in 56 studies (52.3%), and high in 35 studies (32.7%). Primary sources of bias included: Small sample sizes, particularly in pilot studies and case reports (n < 10 in 8 studies), Disease-specific populations limiting generalizability, Lack of polysomnography validation for sleep studies (noted in 35% of sleep studies), Missing data and variable adherence rates (ranging from 31% to 87%), and Reliance on self-reported outcome measures for subjective symptoms. For randomized trials (RoB 2.0), two studies showed moderate risk and five showed high risk of bias, primarily due to lack of blinding, missing outcome data, and COVID-19 pandemic disruptions affecting study protocols (Supplementary File).

## 4. Discussion

This systematic review of 107 studies encompassing approximately 100,000 participants reveals that smart rings have evolved substantially beyond their origins as sleep trackers. The near-equal distribution between sleep (47.7%) and non-sleep applications (52.3%) marks a pivotal transition in the field, demonstrating their emergence as versatile clinical tools capable of continuous physiological monitoring across diverse medical domains.

Our findings demonstrate robust measurement accuracy for core physiological parameters, with HR showing exceptional correlation and HRV compared to gold-standard electrocardiography [37]. These accuracy levels exceed those reported for many wrist-worn devices, likely due to the finger’s superior vascular anatomy and reduced motion artifacts during measurement [7,10]. The clinical significance extends beyond accuracy metrics—smart rings demonstrated predictive capabilities that could transform disease management paradigms. The ability to detect COVID-19 2.75 days before symptom onset [18] and predict IBD flares 7 weeks in advance [50] represents a shift from reactive to proactive healthcare delivery.

The technical architecture underlying smart rings’ measurement capabilities leverages multi-wavelength PPG systems with documented anatomical advantages of finger-based sensing. The Oura Ring Generation 4 employs an 18-path PPG system with green, red, and infrared LEDs, achieving 120% improvement in blood oxygen signal quality compared to previous generations [69]. Contemporary smart rings sample PPG signals at 250 Hz during sleep measurements, enabling detailed heart rate variability analysis [70]. The finger location provides superior signal quality with 95% waveform analyzability versus 67–86% for wrist measurements, attributable to higher vascular density and closer proximity to digital arteries [71]. Advanced signal processing incorporates temporally constrained Independent Component Analysis (cICA) combined with adaptive filtering to remove motion artifacts, achieving mean absolute error of 0.92 bpm during intensive exercise [72,73]. These implementations demonstrate r^2^ = 0.996 correlation with ECG for nocturnal heart rate measurements [70].

Beyond PPG measurements, emerging smart ring architectures demonstrate expanded sensing capabilities through multi-modal integration. Temperature sensors with 0.07 °C resolution enable menstrual cycle tracking and circadian rhythm assessment [43], while 3-axis accelerometers facilitate sleep staging algorithms achieving 91.7–91.8% accuracy against polysomnography gold standards with 421,045 epochs analyzed [20]. The Oura Sleep Staging Algorithm integrates accelerometer, heart rate, heart rate variability, and temperature data through ML, achieving 79% four-stage classification accuracy, approaching the 83% inter-rater agreement among human polysomnography technicians [74]. Critical engineering challenges include contact pressure optimization—sub-optimal pressure significantly degrades wrist PPG accuracy while finger measurements maintain stability [71]—and thermal management to prevent vasoconstriction-induced signal loss. Despite these technical advances, validation remains limited with only 11% of commercial wearables publishing peer-reviewed accuracy data across 249 validation studies encompassing 430,465 participants [75].

The expansion into women’s health applications deserves particular attention. The 96.4% ovulation detection accuracy [45] and ability to predict labor onset within 2 days error at 8 days before delivery [46] could revolutionize reproductive health monitoring. These capabilities, combined with continuous pregnancy monitoring that distinguished outcomes based on temperature patterns [13], suggest smart rings could address critical gaps in women’s health surveillance, particularly in resource-limited settings where access to frequent clinical monitoring is challenging.

The clinical utility of HRV measurement extends beyond these applications. Traditional electrocardiography studies have shown that HRV decreases by 86% in gastric cancer patients and correlates with tumor progression markers [68], suggesting that smart rings’ accurate HRV monitoring capability could potentially enable non-invasive cancer progression surveillance.

Beyond measurement accuracy and predictive capabilities, the successful clinical integration of smart rings faces multifaceted implementation challenges that extend across healthcare systems, providers, and patients. Healthcare infrastructure readiness remains a significant barrier, as most clinical settings lack standardized protocols for incorporating continuous wearable data into existing electronic health record systems and clinical decision-making workflows [1,14]. The volume of data generated by 24/7 monitoring—potentially thousands of data points per patient per day—exceeds the interpretive capacity of current healthcare delivery models designed around episodic measurements [1]. Training requirements for healthcare providers present another layer of complexity, as interpreting longitudinal physiological trends and distinguishing clinically meaningful changes from normal variability requires expertise that transcends traditional vital sign assessment [6,14]. Furthermore, reimbursement frameworks have not evolved to support remote monitoring infrastructure, creating financial disincentives for healthcare systems to invest in integration despite potential long-term cost savings from early disease detection and reduced acute care utilization [2,3]. These systemic barriers must be addressed alongside technological refinement to realize smart rings’ clinical potential [5,6].

While the large aggregate sample size and diversity of applications represent strengths, several critical limitations threaten the generalizability and clinical translation of these findings. The overwhelming dominance of the Oura Ring (72%) creates a concerning single-point dependency that limits cross-platform validation and raises questions about the generalizability of findings to other devices. This concentration mirrors early smartwatch research dominated by Apple Watch studies, potentially creating vendor lock-in effects that could limit clinical adoption [14].

The proprietary nature of algorithms used in 89% of studies represents a fundamental barrier to scientific reproducibility and clinical validation. Without access to underlying computational methods, independent verification becomes impossible, potentially masking device-specific biases or limitations. This algorithmic opacity contrasts sharply with traditional medical devices where measurement principles are transparent and standardized [6].

Population homogeneity poses another significant challenge. Only 35% of studies reported race/ethnicity data, and most participants were young, healthy, educated individuals from high-income countries. Given known variations in PPG signal quality across skin tones and the documented disparities in wearable device accuracy among different ethnic groups [7], this homogeneity severely limits the applicability of findings to diverse clinical populations who might benefit most from remote monitoring.

The documented decline in adherence from 80% at 3 months to 43% at 12 months reveals a critical implementation challenge that transcends technical accuracy. This adherence trajectory, while better than some digital health interventions, remains insufficient for chronic disease management requiring continuous monitoring [68]. The reasons for discontinuation—including comfort issues, battery life constraints, and data interpretation burden—highlight the need for user-centered design improvements and clinical workflow integration strategies [2,12].

Beyond measurement accuracy and clinical validity, human-centered design factors critically influence long-term adoption of smart rings in clinical practice. Ergonomic compatibility with finger biomechanics represents a fundamental determinant of continuous wearability, as the ring form factor must accommodate flexion, extension, and rotational movements during daily activities while maintaining optimal sensor contact [7,10]. Studies in our review documented variable comfort experiences including finger swelling, skin irritation, and sizing challenges across physiological states, with only 35% reporting demographic diversity data to assess device performance across different skin types and ethnicities. Battery life emerged as another critical usability factor, with current devices requiring charging every 4–7 days—a frequency that, while superior to daily-charging smartwatches, still interrupts continuous monitoring and correlates with adherence decline documented in our review [11,12]. The proprietary nature of charging systems and 20–80 min charging durations create dependencies on specific accessories and introduce monitoring gaps during critical periods, while battery degradation over 2–3 year device lifetimes remains unexamined despite relevance for chronic disease management.

Wireless connectivity reliability and user interface design present additional barriers to clinical integration that warrant systematic evaluation. Current smart rings predominantly utilize Bluetooth Low Energy for smartphone synchronization, introducing potential data completeness failures that most studies failed to quantify explicitly [6,14]. Users must maintain device proximity for data transfer, and synchronization failures requiring manual troubleshooting may disproportionately affect older adults or those with limited technical literacy, while reliance on proprietary cloud platforms for algorithm processing creates vulnerabilities during internet outages [1,6]. Healthcare provider interfaces must efficiently present thousands of daily data points within existing clinical workflows, yet no studies systematically evaluated clinician burden or integration pathways with electronic health records [1,14]. The tension between comprehensive data presentation and cognitive overwhelm requires careful navigation, as users must interpret probabilistic predictions and distinguish actionable alerts from normal physiological fluctuations without standardized communication frameworks [6]. Future research should employ formal usability testing methodologies (e.g., System Usability Scale, NASA Task Load Index), conduct longitudinal assessments of device comfort beyond 6 months, and engage target clinical populations in user-centered design research to ensure solutions address real-world constraints alongside technical validation [12,14].

Smart rings represent components within a broader connected health ecosystem, necessitating consideration of interoperability and integration challenges that extend beyond individual device performance. Current smart ring implementations operate predominantly as isolated data collection systems with limited bidirectional communication capabilities with other medical devices or electronic health records (EHRs) [1,6]. The proprietary nature of data formats, application programming interfaces, and cloud platforms identified in our review creates significant barriers to seamless integration with existing clinical information systems [6,14]. Standardized data exchange protocols—such as Fast Healthcare Interoperability Resources (FHIR)—could enable automatic transmission of physiological data from smart rings into EHRs, allowing clinicians to view longitudinal trends alongside laboratory results, imaging, and other clinical data within unified interfaces [1]. However, regulatory frameworks, data security requirements, and vendor business models currently impede widespread adoption of open interoperability standards [14]. Multi-device integration scenarios, where smart rings complement continuous glucose monitors, implantable cardiac devices, or medication adherence sensors, could provide synergistic insights exceeding individual data streams, yet technical and regulatory infrastructure for such integration remains underdeveloped [5,6].

The evolution toward closed-loop systems, where smart ring data automatically triggers clinical interventions without human intermediation, represents a transformative but largely unrealized frontier. Conceptually, smart ring detection of physiological deterioration—such as the 2.75-day advance COVID-19 detection demonstrated in our review—could automatically initiate telemedicine consultations, dispatch community health workers, or trigger medication adjustments through connected delivery systems [1,5]. For chronic disease management, automated alerts based on heart rate variability decline could prompt medication titration for heart failure patients, while temperature elevation patterns could trigger antibiotic delivery for immunocompromised individuals [2,5]. However, implementing such closed-loop systems requires addressing substantial safety, liability, and validation challenges [1,14]. False positive rates that might be acceptable for informational alerts become unacceptable when triggering medical interventions, necessitating rigorous validation across diverse populations and clinical scenarios not represented in current studies [6,14]. Regulatory pathways for autonomous medical decision-making devices remain unclear, and medicolegal frameworks for attributing responsibility when algorithms initiate inappropriate interventions require development [14]. Furthermore, patient autonomy and informed consent become complex when continuous monitoring systems make automated treatment decisions, particularly for populations with limited health literacy or technical expertise [2,6]. Future research should systematically evaluate technical architectures for secure, standards-based interoperability, develop validation frameworks for closed-loop intervention systems, and engage stakeholders including patients, clinicians, and regulators in defining appropriate boundaries for autonomous medical decision-making [1,5,14].

Our findings align with and extend previous systematic reviews of wearable devices in clinical settings [1,2,9]. While prior reviews focused primarily on measurement validation [9], our analysis reveals the transition toward clinical outcome prediction and intervention guidance. The balanced distribution between sleep and non-sleep applications contrasts with earlier reviews that emphasized sleep as the primary use case, confirming the technology’s maturation beyond its initial consumer wellness focus [4,5].

The convergence of smart ring technology with artificial intelligence and ML represents a transformative opportunity for enhancing clinical utility and personalization. Current applications rely primarily on population-based algorithms and fixed thresholds, but individual physiological baselines vary substantially across age, sex, fitness level, and underlying health conditions [1,7,10]. ML approaches could enable dynamic, personalized baseline establishment and context-aware anomaly detection that accounts for circadian rhythms, activity patterns, and seasonal variations [5,9]. The integration of multi-modal data streams—combining smart ring physiological signals with other wearable sensors, electronic health records, and patient-reported outcomes—could enhance predictive accuracy beyond what any single data source achieves in isolation [1,2,6]. Furthermore, federated learning architectures could enable algorithm refinement across diverse populations while preserving individual privacy, addressing both the algorithmic transparency concerns and population homogeneity limitations identified in our review [6,14]. The miniaturization trends enabling ring form factors continue to advance, with emerging biosensors capable of measuring additional parameters such as continuous glucose, cortisol, and inflammatory biomarkers showing promise in early-stage development [5,11]. These technological advances, combined with improved battery efficiency and wireless charging capabilities, could overcome current adherence barriers related to comfort and usability [10,12].

Several priority areas emerge for advancing smart ring clinical applications. First, multi-vendor validation studies using standardized protocols are essential to establish device-agnostic clinical guidelines. Second, algorithm transparency through open-source initiatives or regulatory requirements could enhance scientific rigor and clinical trust [6]. Third, purposeful recruitment of diverse populations, particularly those with chronic conditions and from underrepresented groups, is critical for ensuring equitable healthcare applications [3].

The integration of smart rings into clinical workflows requires careful consideration of data management, interpretation, and liability issues. The volume of continuous data generated presents both opportunities and challenges for healthcare systems already struggling with information overload [1].

The ethical implications of continuous physiological monitoring through smart rings warrant careful consideration as the technology approaches clinical deployment. Continuous data collection raises fundamental questions about patient autonomy, informed consent, and the potential for surveillance that extends beyond therapeutic benefit [1,6]. Privacy and data security concerns become paramount when sensitive health information flows continuously from personal devices through commercial platforms to healthcare providers, creating multiple points of vulnerability for data breaches or unauthorized access [14]. The opacity of proprietary algorithms compounds these concerns, as patients and clinicians cannot fully understand how raw physiological signals are transformed into health metrics and predictions [6]. Regulatory frameworks must evolve to address these unique challenges while fostering innovation [2,3]. Looking forward, the integration of artificial intelligence for personalized baseline establishment and anomaly detection holds promise for enhancing clinical utility [5,9], but requires rigorous validation across diverse populations to ensure equitable benefit distribution [3,7]. Establishing international standards for data interoperability, algorithm transparency, and privacy protection represents a critical prerequisite for responsible clinical adoption [6,10,11].

This review has several limitations. The heterogeneity of populations, devices, and outcomes precluded meta-analysis, limiting quantitative synthesis. Publication bias toward positive findings may overestimate device performance, particularly given the prevalence of industry-sponsored studies. The rapid evolution of smart ring technology means newer devices and algorithms may have addressed some identified limitations.

## 5. Conclusions

Smart rings demonstrate accuracy and predictive capabilities across clinical domains, with equal sleep/non-sleep distribution confirming evolution from wellness devices to clinical tools. However, device diversity limitations, algorithmic transparency, population representativeness, and long-term adherence challenges require attention through rigorous research and user-centered design to realize potential for enhanced clinical care.

From an engineering standpoint, next-generation smart ring development must address critical challenges including sensor miniaturization while maintaining signal quality, extended battery life through energy harvesting and ultra-low-power designs, and advanced signal processing for motion artifact rejection during intensive activity. Materials engineering advances in biocompatible, hypoallergenic substrates would address comfort limitations affecting long-term adherence. Multi-modal sensor integration incorporating electrochemical biosensors for continuous metabolite monitoring could expand clinical utility beyond cardiovascular applications, though miniaturization and power constraints present substantial barriers. Ultimately, standardized hardware interfaces and open-architecture designs enabling third-party algorithm development would address algorithmic opacity and vendor lock-in, requiring collaboration between manufacturers, regulators, and researchers to establish industry-wide technical specifications that balance innovation with clinical validation requirements.

## Figures and Tables

**Table 1:** Searching strategy to find the relevant articles.

Database: MEDLINE (Through PubMed)
#1 “smart ring”[tiab] OR “ring-shaped wearable”[tiab] OR “finger-worn”[tiab]: 61 #2 “Oura”[tiab] OR “Circul”[tiab] OR “CART-I”[tiab]: 143 #3 #1 OR #2: 188 #4 #3 AND English[Lang]: 186
Database: Embase
#1 ‘smart ring’:ab,ti,kw OR ‘ring-shaped wearable’:ab,ti,kw OR ‘finger-worn’:ab,ti,kw: 549 #2 ‘Oura’:ab,ti,kw OR ‘Circul’:ab,ti,kw OR ‘CART-I’:ab,ti,kw: 267 #3 #1 OR #2: 790 #4 #3 AND ([article]/lim OR [article in press]/lim OR [review]/lim) AND [English]/lim: 371
Database: Cochrane Library
#1 ‘smart ring’:ab,ti,kw OR ‘ring-shaped wearable’:ab,ti,kw OR ‘finger-worn’:ab,ti,kw: 32 #2 ‘Oura’:ab,ti,kw OR ‘Circul’:ab,ti,kw OR ‘CART-I’:ab,ti,kw: 54 #3 #1 OR #2: 82
Database: Web of Science
#1 ab = (“smart ring” OR “ring-shaped wearable” OR “finger-worn”): 76 #2 ab = (“Oura” OR “Circul” OR “CART-I”): 118 #3 #1 OR #2: 186

## Data Availability

All the data are accessible and available upon request by corresponding author. Access to data: All investigators have access to the final dataset.

## Supplementary Materials

The following supporting information can be downloaded at: https://www.mdpi.com/article/10.3390/biomimetics10120819/s1, Table S1: Clinical summary of the included sleep studies; Table S2: Clinical summary of the included non-sleep studies; Table S3: Risk of bias evaluation (ROBINS-I assessment tool) for sleep studies; Table S4: Risk of bias 2.0 (ROB 2.0 tool) for sleep studies; Table S5: Risk of bias evaluation (ROBINS-I assessment tool) for non-sleep studies; Table S6: Risk of bias 2.0 (ROB 2.0 tool) for non-sleep studies.
