# Supplementary material for: Smart Ring in Clinical Medicine: A Systematic Review
Source: Biomimetics (Basel). 2025 Dec 5;10(12):819. doi: 10.3390/biomimetics10120819 (PMC12730986; doi:10.3390/biomimetics10120819)
Supplement: Supplementary file 1 [file biomimetics-10-00819-s001.zip › biomimetics-3979884-supplementary.pdf]

Supplementary Table S1. Clinical summary of the included sleep studies

Study/year	Nationality	Study Design	Population (Sample Size, age: mean ± standard deviation)	Smart Ring Device	Clinical Purpose / Primary outcome	Results / clinical performance	Limitation	Overall risk of bias
Bigalke JA et al., 2025[1]	US	Observational	24 men collegiate baseball players (age: 21 ± 1 years)	Oura	Sleep-power performance relationship	≥7h sleep correlated with peak power	Observational, no causality	Moderate
Ng ASC et al., 2025[2]	Singapore	Observational	119 university students (55 men, 22.54 ± 1.74)	Oura	Sleep-blood glucose relationship	Longer sleep associated with lower glucose variability	Healthy participants only	Moderate
Meira E Cruz M et al., 2025[3]	China	Cross-sectional	164 patients suspected of having sleep-disordered breathing	Circul	Sleep breathing disorder detection	Strong polysomnography correlation (R^2=0.9012)		Low
Herberger S et al., 2025[4]	Germany	Cross-sectional	45 participants (20 women)	Oura, SleepOn, Circul	Sleep measurement vs polysomnography	Oura sleep metrics accuracy 85.03%, sleep staging accuracy 53.18%	Single night, clinical population only	Moderate
Ju E et al., 2025[5]	US (Korean American)	Observational	11 patients with dementia & 11 caregivers	Oura	Sleep quality assessment in dementia patients and family caregivers	Sleep parameters of patients with dementia were significantly correlated with those of caregivers.	Small sample	Moderate
Barba E et al., 2025[6]	Spain	Observational	20 Female professional soccer players	Oura	Sleep quality and quantity analysis using principal component analysis	A combination of both quantity and quality sleep metrics is recommended	No training load consideration, uncertainty in sleep staging	Moderate
Willoughby AR et al., 2025[7]	Global	Observational	57 240 Oura Ring users (32166 women , men: 46.04 ± 13.03, women : 42.88 ± 13.08)	Oura	Travel-related sleep disruption	Sleep timing took >15 days to recover	No control (for travel purpose)	Low

Montesino-Goicolea S et al., 2025[8]	US	Secondary data analysis of a pilot study double-blinded, placebo-controlled, randomized parallel group clinical trial	33 chronic musculoskeletal pain patients (26 women 68 ± 7.4)	Oura	Subjective vs objective sleep in chronic musculoskeletal pain patients	Pittsburgh Sleep Quality Index-Oura correlation for duration (r=0.71)	Small sample, no polysomnography validation	High
Vatne E et al., 2025[9]	US	Observational	97 College student-athletes (52.57% women)	Oura	Effects of daytime floatation-restricted environmental stimulation therapy on nocturnal cardiovascular physiology, sleep, and subjective recovery	Reduced resting heart rate post-float	No control group	Moderate
Norful AA et al., 2025[10]	US	Prospective pilot trial	25 nurses (45.2 ± 11.6 years, 16 White, 3 Asians)	Oura	longitudinal physiologic stress measurement and sleep quality interventions using sleep aids	Sleep latency decreased (p=0.03)	Single-arm design, small sample	High
Qin S et al., 2025[11]	Singapore	Cross-sectional	773 community-dwelling older adults of ages 65–80 years,	Oura	Sleep-cognition association	Sleep regularity correlated with executive function	Survivorship bias, no obstructive sleep apnea measures	Moderate
Soon CS et al., 2025[12]	Singapore	Observational	638 university freshmen (20.3 ± 1.3 years; women 51.7 %)	Oura	Academic schedule effects on sleep	Bedtimes were 71.76 min late, 42.6% woke after class start	Single university, decreasing participation	High
Smith ES et al., 2025[13]	Australia	Randomized crossover	20 endurance athletes (10 women, 35 ± 8 years)	Oura	Energy availability effects on sleep	24-h of exercise-induced low energy availability extended total sleep time, while diet-induced low energy availability reduced mean overnight heart rate.	Participants taking oral contraceptive pill only	Moderate
Moen LV et al., 2025[14]	Norway	Cross-sectional	60 shift workers (mean 28 years, 74.5% men)	Oura	Validation of the Norwegian version of the Munich ChronoType Questionnaire	High correlations (r>0.6) with Oura measurements	Black-box algorithms (inaccessible raw data of wearable	Moderate

							device and lack of control), work restrictions	
Singh A et al., 2025[15]	US	Case series	14 obstructive sleep apnea patients (8 women, 62.2 ± 9.2)	Oura	Changes in wearable sleep technology metrics during hypoglossal nerve stimulation	Higher hypoglossal nerve stimulation amplitude decreased TST), wake time after sleep onset (p=0.002)	Small sample, variable data quality	High
Font R et al., 2025[16]	Spain	Observational	13 elite handball players (13 men, 28.4 ± 3.7 years)	Oura	Sleep during competitive season	No physiological alterations, moderate differences in total time in bed between home matches and travel days	Single team, couldn't wear during matches	Moderate
Debbiche I et al. 2025[17]	US	Observational (Secondary Analysis)	90 Gynecologic surgery patients (54.8 ± 12.8 years; 63.3% with cancer)	Oura	Preoperative sleep efficiency impact on postoperative outcomes / 30-day complications, opioid use	Better sleep efficiency significantly reduced postoperative complications and opioid requirements; High sleep efficiency patients had 3-fold lower complication rates	Single institution, No symptom burden assessment	High
Donahue CC et al. 2025[18]	US	Case-control	18 Collegiate athletes (19.3 ± 1.3 years, 9 concussed, 9 controls)	Oura	Acute sleep stage changes post-concussion within 72 hours	Concussed athletes showed significantly increased nighttime wakefulness and reduced deep sleep. Sleep architecture disruption evident in acute phase	Small sample, No pre-injury baseline data, Single institution	Moderate
Fein T et al. 2025[19]	US	Crossover Pilot	15 Healthy adults (22.7±5.7 years; 66.7% men)	Oura	Fluid intake effects on sleep quality and duration changes	Strong positive relationship between water intake and REM sleep in dehydrated individuals; Adequate hydration associated with better sleep quality	Small sample, pilot study design, insufficient fluid reduction (5%), short monitoring periods	High
Massar SAA et al., 2024[20]	Singapore	Observational	96 medical interns	Oura	Sleep, well-being, and cognition comparison between Float vs call schedule	More regular and better-quality sleep on a float	Observational design, selection bias	Moderate

						schedule than on a call schedule		
Robbins R et al., 2024[21]	US	Cross-sectional	35 participants (aged 20–50 years, 20 women) without a sleep disorder	Oura	Sleep tracking performance validation against polysomnography	95% sleep detection sensitivity	Single night, healthy only	Low
Labbaf S et al., 2024[22]	US	Observational	21 university students (13 Asians, 14 women)	Oura	Physiological and emotional assessment of college students during the 2020 COVID-19 lockdown	85% engagement with device	Small sample, specific period	Moderate
Viswanath VK et al., 2024[23]	US	Observational	33,152 individuals (44.4 ± 12 years, 19,792 men)	Oura	Sleep phenotype dynamics	13 phenotypes identified, transitions differ by health status	Self-reported conditions	Moderate
Fudolig MI et al., 2024[24]	US	Cross-sectional	599 university students	Oura	Explore the connection of heart rate patterns and mental health	Later heart rate nadir predicted anxiety/depression	University students only	Moderate
Armitage BT et al., 2024[25]	UK	Randomized controlled pilot study	101 general population adults (31.0 ± 11.2 years, 73% women)	Oura	Sleep app effectiveness	No significant app effect on sleep quality	COVID-19 disruption	High
Hausenblas HA et al., 2024[26]	US	Double-blind, placebo-controlled, parallel-arm randomized controlled study	80 adults with nonclinical insomnia symptoms (45.49 ± 6.00, 63 women)	Oura	Magnesium-L-threonate supplementation effects on sleep quality and daytime functioning	Significant improvements in multiple sleep parameters	Short administration duration (21 days)	Low
Breus M et al., 2024[27]	US	A quasi-experimental intervention study	39 adults (12 men, 45.29 ± 6.12) with nonclinical insomnia	Oura	Examine the effectiveness of a pressure-releasing medium-firm grid mattress on sleep and health outcomes (e.g., mood, pain, daytime fatigue)	Significant improvements in multiple sleep metrics	No control group, not randomized	High
Willoughby AR et al., 2024[28]	Singapore	Cross-sectional	63 participants (36 women) aged 20-68	Oura (+4 devices)	Sleep/wake transition detection	High detection accuracy (0.82) for sleep onset	Lab setting	Moderate

Svensson T et al., 2024[29]	Japan	Cross-sectional	96 generally healthy Japanese men and women aged between 20 and 70 years	Oura	Performance validation with Oura sleep staging algorithm 2.0 compared to multi-night ambulatory polysomnography	Sensitivity of 94.4 %–94.5 %, Specificity of 73.0 %– 74.6 %	Hotel setting, proprietary algorithm	Moderate
Kainec KA et al., 2024[30]	US	Cross-sectional	53 healthy young adults (31 women, 22.5 ± 3.5 years)	Oura (+4 devices)	Five device accuracy evaluation for sleep tracking	Intraclass correlation coefficient 0.77 for total sleep time, poor for sleep stages	Single night measurement	Moderate
Kubala AG et al., 2024[31]	US	Observational	853 US Navy shipboard personnel	Oura	Operational feasibility and acceptability of commercial wearable devices for sleep monitoring	Feasible for shipboard use (individual use rates were 71 ± 38%)	Specific military population	Moderate
Ong JL et al., 2024[32]	Singapore	Cross-sectional	66 adults aged 20-68 years (40.2 ± 15.7) years; 29 men; ethnically composed of Chinese: 85%, Indian: 8%, Malay: 1%, Others: 6%)	Oura	Multi-device sleep tracking comparison (electroencephalography-based vs consumer devices)	Good agreement for basic sleep metrics	Lab setting, healthy only	Low
Hall LS et al., 2024[33]	New Zealand	Observational	33 dairy farm workers	Oura	Quantifying the sleep quantity and quality of farmers over calving period	Total sleep time averaged 6h 15min, below recommended	Small sample, rings worn 57% of time	High
Ou YH et al., 2023[34]	Singapore	Observational	129 hypertension patients (114 men, 60.5 ± 8.4 years)	Belun	Obstructive sleep apnea diagnosis in high-risk patients	Sensitivity 77.6% specificity 85.3%	Single center, mostly men enrolled	Moderate
Lee T et al., 2023[35]	Korea	Cross-sectional	75 participants from a tertiary hospital and a primary sleep-specialized clinic	Oura	Multi-device sleep tracking comparison	Variable performance across devices (sleep stage accuracy 0.5427 for Oura)	Single night measurement	Moderate
Vanhanen J et al., 2023[36]	Finland	Randomized, sham-controlled trial	32 patients with complex regional pain syndrome	Oura	Repetitive transcranial magnetic stimulation effects on sleep in complex regional pain syndrome	Sleep quality improvements with repetitive transcranial magnetic stimulation	Small sample	Moderate

Nauha L et al., 2023[37]	Finland	Cross-sectional	108 adults (61 women, 33.1 ± 0.4)	Oura	Sleep period agreement with self-reported diary	Intraclass correlation coefficients >0.81 for bedtimes and wake times	Single cohort, one age group	Moderate
Chinoy ED et al., 2023[38]	US	Cross-sectional	16 healthy young adults (6 male, 10 women; 26.6 ± 4.6 years)	Oura	Daytime sleep tracking performance	Total missed daytime sleep episodes: 6%	Small sample, self-report reference	Moderate
Shuster AE et al., 2023[39]	US	Observational	76 healthy young women participants	Oura	Sleep-mood interaction across menstrual cycle	Good sleep buffered mood during menses	No hormone measurements, healthy only	Moderate
de Vries HJ et al., 2023[40]	Netherlan ds	Observational	Eight police officers	Oura	Sleep and resting heart rate variability as an outcome of and predictor for subjective stress measures	Total sleep time negatively predicted stress in 3/8 participants	Very small sample, COVID-19 context	High
Grandner MA et al., 2023[41]	US	Cross-sectional	36 healthy adults	Happy Ring (vs Oura)	Multi-sensor smart ring evaluation	93% sensitivity, 83% specificity for sleep detection (compared to in-lab polysomnography)	Small sample, healthy adults only	Low
Chinoy ED et al., 2022[42]	US	Cross-sectional	21 healthy young adults (12 women, 9 men)	Oura	Daytime sleep tracking performance	Low time in bed bias. sensitivity (for sleep): 0.94 and specificity (for wake): 0.41	Young healthy adults only	Moderate
Zhao R et al., 2022[43]	China	Cross-sectional	207 apnea patients	Circul	Obstructive sleep apnea detection	apnea–hypopnea index ≥5 events/hour: 87% sensitivity, 83% specificity, AUC 0.929	Single night	Low
Mousavi ZA et al., 2022[44]	US	Observational	20 College student participants (women: 13)	Oura	Sleep-affect associations during COVID-19 pandemic	Fluctuating sleep patterns are associated with affect dynamics at the daily and long-term scales.	Small sample & homogenous age: mean 19.80 ± 1.0 years	Moderate
Rentz LE et al., 2022[45]	US	Observational	12 women soccer players	Oura	Photobiomodulation therapy effects on sleep	40 min total sleep time reduction with photobiomodulation therapy (p=0.0006)	Small sample, no control group	Moderate

Ghorbani S et al., 2022[46]	Singapore	Cross-sectional	58 healthy East Asians (32 men, 37.1 ± 13.03 years)	Oura	Algorithm comparison (Gen2 vs Gen3)	Sleep stage classification accuracy: Gen3: 92.6% vs Gen2: 89.6%	Healthy participants only, East Asian only	Moderate
Henriksen A et al., 2022[47]	Norway	Cross-sectional	21 healthy adults (12 men, 33 ± 14 years)	Oura	Activity and sleep measurement	Sleep duration r=0.82 with diary, resting heart rates r=0.9 with Actiheart accelerometer	Small sample, no gold standard	Moderate
Roberts DM et al., 2020[48]	US	Cross-sectional validation	8 participants	Oura	Machine learning classifiers for sleep detection (Data from multisensor wearables (Apple Watch and Oura Ring) were compared to data available from electrocardiography and a wrist actigraphy to evaluate the quality and utility of heart rate and motion data)	Sensitivity 0.963, specificity 0.410	Very small sample (n=8)	High
Stone JD et al., 2020[49]	US	Cross-sectional	5 healthy adults, 2 men and 3 women (98 nights)	Oura	Evaluate accuracy vs EEG-based device	Total sleep time mean absolute % error: 7.39%, poor sleep staging accuracy	Very small sample (n=5), light skin tones only	High
Chaudhry FF et al., 2020[50]	US	Cross-sectional	21 participants (11 women; 10 men)	Oura	Sleep metrics correlation with cognitive/psychological measures	Moderate device correlations (0.37-0.51 for total sleep duration)	No gold standard, missing data	High
Asgari Mehrabadi M et al. 2020[51]	Finland	Cross-sectional	45 healthy adults (23 women, 33.1 ± 6.4 years)	Oura	Sleep parameter validation against medical-grade actigraphy	Strong agreement for total sleep time; Moderate agreement for wake episodes and sleep efficiency	No polysomnography comparison, healthy adults only, cannot validate sleep stages	Moderate

AUC, area under the curve

Supplementary Table S2. Clinical summary of the included non-sleep studies

Study/year	Nationality	Study Design	Population (Sample Size)	Smart Ring Device	Clinical Purpose / Primary outcome	Clinical performance	Limitation	Overall risk of bias
Borelli JL et al., 2025[52]	US	Observational	28 undergraduate students (13 women, 19.96 ± 1.23 years)	Oura + Samsung watch + smartphone	Depression detection	Light gradient boosting machine model: F1-score 0.744	Small sample, specific population, limited generalizability	Moderate
Dupuit M et al., 2025[53]	France	Case report	2 Olympic female swimmers	Oura	Heart rate variability across menstrual cycle in athletes	Heart rate variability patterns varied with menstrual phases and training	Case report design, n=2	High
Hadid A et al., 2025[54]	Canada	Prospective controlled trial	55 Healthy adults (28 men, mean 30 years)	Oura, smart watch, electrocardiography etc.	Predict systemic inflammation post-live attenuated influenza vaccine	AUC: 0.89 (24h-tolerance); outperformed symptom-based detection	Small sample, homogenous population, short follow-up	Moderate
Hanish RR et al., 2025[55]	US	Observational	23 obsessive-compulsive disorder patients (14 with Oura data)	Oura	Correlation between activity and neural artifacts (vs Medtronic Percept device)	Significant correlation (p<0.021) between metabolic equivalent values and neural activity	Small subset with Oura data, limited to obsessive-compulsive disorder patients	Moderate
Hirten IP et al., 2025[56]	US	Observational	309 inflammatory bowel disease patients	Oura or Apple Watch or Fitbit	Predict inflammatory bowel disease flares	Heart rate variability patterns differed between flare/remission; changes 7 weeks before flares	Disease-specific population	Moderate
Ortiz A et al., 2025[57]	US	Observational	127 bipolar disorder patients (82 women, 39.2 ± 12.6 years)	Oura	Predict depressive episodes through activity variability	Activity variability detected depression 7.0 days earlier than sleep; 79% sensitivity	Disease-specific population	Moderate

Reithe H et al., 2025[58]	Multinational	Observational	15 Parkinson's disease patients and 16 controls	Oura vs. Empatica E4 vs. Fitbit Sense	Device comparison for Parkinson's disease monitoring	Limited cross-device compatibility; Oura showed poor continuous HR/movement recording	Disease-specific, device limitations	Moderate
Rim J et al., 2025[59]	US	Observational	9 Pregnant women (28.8 ± 4.7 years)	Oura	Physical activity effect on heart rate variability during pregnancy	Physical activity provided protective effects against autonomic dysregulation	Observational design, small sample	High
Thigpen N et al., 2025[60]	US	Cross-sectional	1155 ovulatory menstrual cycles from 964 women participants	Oura	Ovulation detection validation	96.4% detection rate; 1.26 days error vs calendar's 3.44 days; 3-fold improvement	Limited to ovulatory cycles only	Low
van Steijn NJ et al., 2025[61]	Netherlands	Observational	25 patients undergoing ventricular arrhythmias procedures (10 women, 54.4 ± 16.9 years)	CART-I	Detect ventricular arrhythmias	Overall sensitivity 94% (Ventricular fibrillation 100%, ventricular tachycardia 90%); intraclass correlation coefficiency 0.998 for duration	Controlled setting only, small sample	Low
Zakaria H et al., 2025[62]	UAE	Case report	1 patient (men), early 40s with type 2 diabetes	Oura	Remote cardiac monitoring	Heart rate variability improved from 15.2 to 32.8 ms over 2 months	Single case report	High
Morimoto M et al., 2024[63]	US	Observational	37 orthopedic surgery patients (17 women, median 58-59 years)	Oura	Predict postoperative pain outcomes	Accuracy 70.0%, F1-score 0.769, AUC 0.762 for per-patient predictions	Small sample, single center, non-blinded	Moderate
Mastrototaro JJ et al., 2024[64]	US	Open enrollment, single-center, single-blinded	11 healthy volunteers (6 men, ages 22-34)	Evie	SpO2 accuracy in hypoxia (70-100% SaO2)	root mean square error 2.1%; met FDA requirements (<3.5% RMSE)	Small sample, healthy young adults only, controlled conditions	Moderate
Mason et AE al., 2024[65]	US	Observational	20,880 individuals (53% men, 46.9 ± 12.6 years)	Oura	Temperature patterns and depression	Explored temperature-depression associations	Limited details available	Moderate

Liang T et al., 2024[66]	Singapore	Cross-sectional	114 participants (92 younger, 22 older, 50 men, 28 ± 15.8 years)	Oura	Heart rate variability validation against electrocardiography	High correlation (r>0.9) with 80% validity threshold; 30-min averaging improved accuracy	Age-related accuracy differences	Low
Kim J et al., 2024[67]	Korea	Cross-sectional	89 healthy adults (40.1 ± 12.0 years)	CART-I	Blood pressure measurement accuracy	Mean difference: SBP 0.16 ± 5.90 mmHg, DBP -0.07 ± 4.68 mmHg; r=0.94-0.95	Small sample, limited populations, single measurement	Low
Keeler Bruce L et al., 2024[68]	US	Observational	120 pregnancies (97 full-term, 23 early loss)	Oura	Complete pregnancy recording	Clear physiological trajectories identified; early loss showed temperature deviations	Observational design	Low
Kasl P et al., 2024[69]	US	Observational	16794 participants	Oura	Syndromic surveillance for fever detection	Demonstrated population health monitoring capabilities	Limited clinical validation	Moderate
Ibrahim AH et al., 2024[70]	US	Cross-sectional	17 regular exercisers	Oura or Whoop band	Exercise recovery experiences	Users modified training and lifestyle based on readiness scores	Small sample	Moderate
D'Adamo CR et al., 2024[71]	US	Pilot intervention	25 healthy adults	Oura	Electromagnetic field protection device effects on health/cognition	Improved heart rate variability, sleep quality, cognitive function with electromagnetic field protection	Small sample, placebo effects possible	Moderate
Alzueta E et al., 2024[72]	US	Observational	117 women (67 reproductive stage , 53 late reproductive to menopause transition)	Oura	Menstrual cycle tracking	96 participants showed oscillatory temperature patterns, heart rate lowest during menses	Single cycle monitoring, no hormonal validation	Moderate
Basavaraj C et al., 2024[73]	US	Observational	91 pregnant women (54 spontaneous labors)	Oura	Deep learning model for labor onset prediction	Autoencoder long short-term memory model: <2 days error at 8 days before labor; 79% accuracy within 4.6-day window	Small sample, proprietary metrics	Moderate
Peterson NE et al., 2024[74]	US	Observational	15 women (45.53 ± 9.2 years)	Oura vs. Apple Watch, Bellabeat	Physical activity motivation	Oura worn 20.4 ± 4.7 hours/day (highest); ranked second for motivation after Apple Watch	Small sample, short duration	Moderate

				Leaf Urban				
Phipps J et al., 2024[75]	US	Observational	Over 8000 participants	Oura	COVID-19 early detection	AUC 0.777; predicted positive cases 4.1 days before test results	Self-reported testing	Moderate
Ryan JM et al., 2024[76]	US	Case study	73 COVID-19 patients	Oura	COVID-19 physiological manifestations	Information theory approaches correlated physiological changes with symptoms	Limited sample size	Moderate
Sharifi-Heris Z et al., 2024[77]	US	Observational	15 Hispanic pregnant women (31.7 ± 3.89 years)	Oura	Feasibility of continuous monitoring during pregnancy/postpartum	>80% adherence until late pregnancy; decreased to ~31% postpartum	Small sample, specific ethnic population	Moderate
Liew SJ et al., 2023[78]	Singapore	Randomized controlled trial (parallel two-arm, single-center, nonblinded)	56 Asian women with previous gestational diabetes (35.8 ± 3.7 years)	Oura	Diabetes prevention through lifestyle intervention	Lifestyle modification supported by continuous monitoring	Study ongoing at time of review	Moderate
Kristiansson E et al., 2023[79]	Sweden	Cross-sectional	32 healthy adults (17 women, 29.6 ± 5.2 years)	Oura	Validate step count and energy expenditure	Strong correlations (r=0.93 lab, r≥0.76 free-living)	Small sample, limited to healthy adults aged 20-40	Moderate
Björkman K et al., 2023[80]	Finland	Cross-sectional	30 preterm-born (19 women, 24.4 ± 0.9 years) & 16 full-term young adults (11 women, 24.0 ± 1.0 years)	Oura	Heart rate variability assessment in preterm-born adults	No significant differences in cardiac autonomic function. Men preterm showed trend toward lower heart rate variability	Small sample, single center, limited generalizability	Low
Balsam D et al., 2023[81]	US	Observational	20 pregnant women (29.45 ± 3.27 years, 16 White, 4 Hispanic, 2 Asians)	Oura	Evaluates mindfulness app impact on stress/anxiety (physiological measures reflective of stress (heart rate variability and sleep) were collected via the Oura Ring	Significant reductions: stress (p=0.005), anxiety (p=0.01), pregnancy anxiety (p<0.0001)	No control group, small homogenous sample, self-selection bias	High

Bruce LK et al., 2023[82]	US	Observational	600 participants (300 women, 49.4 ± 16.4 years)	Oura	Analyze temperature variability by biological sex	No evidence females more variable than males; majority of variance within sex categories	Limited to temperature only, single device type	Moderate
Croghan IT et al., 2023[83]	US	Pilot intervention	10 Healthcare professionals (6 men, 6 White, 3 Asians, 39.7 ± 5.55 years)	Oura	Stress resilience program during pandemic (sleep check with Oura)	Improved resilience, reduced stress during COVID-19	Small sample, no control group, short duration	High
Erickson EN et al., 2023[84]	US	Observational	118 pregnant participants (32.6 ± 4.1 years)	Oura	Predict labor onset relative to estimated delivery date	A boosted random forest model: AUC 0.71; 79% accuracy within 4.6-day window at 7 days before labor	Limited to healthy, low-risk pregnancies; mostly White participants	Moderate
Jafarlou S et al., 2023[85]	US	Observational	20 college students	Oura	Predict next-day affect/mood	Random forest ~78% accuracy positive affect; ~72% stress prediction	Small sample, high dropout, COVID-19 impact	High
Nolasco HR et al., 2023[86]	US	Observational	31 Japanese adults	Oura	Device adherence study	87% compliance rate over 30 days; 15-19 hours daily wear	Limited details on population	Moderate
Padmanabha A et al., 2023[87]	US	Cross-sectional	20 healthy adults	Self-developed	Scratch intensity measurement	89.98% accuracy scratch detection	Prototype device, limited validation	Moderate
Sanai F et al., 2023[88]	Australia	Observational	14 adult participants with type 2 diabetes	Self-developed bioimpedance ring	Non-invasive glucose monitoring	Accuracy within ±20 mg/dL for 83.6% of measurements	Prototype device	Moderate
Shiba SK et al., 2023[89]	US	Observational	91 healthcare workers (54 women, 36 ± 13 years)	Oura	Adherence: wearable vs surveys for COVID-19 detection	Wearable adherence 87.8 ± 11.6% vs survey 63.8 ± 27.4%	Small sample, early pandemic	Moderate
Mason AE et al., 2022[90]	US	Observational	63,153 participants; 73 COVID-19 positive	Oura	COVID-19 detection	82% sensitivity, 63% specificity; 2.75 days early detection	Algorithm training on small positive sample	Moderate

Mason AE et al., 2022[91]	US	Observational	1,179 vaccine recipients	Oura	COVID-19 vaccine response prediction	Temperature/HR changes correlated with antibody response	Observational design	Low
Alzueta E et al., 2022[92]	US	Observational	26 healthy women (24.4 ± 1.1 years)	Oura	Track physiological changes across menstrual cycle	HR and temperature showed biphasic pattern; No significant sleep changes across cycle phases	Small sample, single cycle per participant, no hormone measurements	Moderate
Cao R et al., 2022[93]	Finland	Cross-sectional	35 healthy individuals (19 women, 32.3 ± 6.4 years)	Oura	Validate heart rate and heart rate variability accuracy during sleep	High Pearson correlation coefficient with heart rate and root mean square of successive differences between normal heartbeats	Limited to nocturnal measurements, healthy participants only	Low
Conroy B et al., 2022[94]	US	Observational	9381 United States Department of Defense personnel (491 COVID-19 positive)	Oura + Garmin	Early COVID-19 infection prediction	AUC 0.82; 60% sensitivity; 2.3 days lead time before testing	Required 48% data capture, symptomatic cases only	Moderate
de Vries H et al., 2022[95]	Netherlands	Observational	68 police officers	Oura	Heart rate variability trends association with stress/somatization	Heart rate variability fluctuations predicted stress and somatization changes	Specific occupation, generalizability limited	Moderate
Fonseka RD et al., 2022[96]	Australia	Case report	1 spine surgery patient	Oura	Monitor recovery after spine surgery	Documented physiological recovery patterns	Single case, not generalizable	High
Jimah T et al., 2022[97]	US	Case report	2 pregnant women	Oura	COVID-19 monitoring during pregnancy	Detected COVID-19 related physiological changes	Case report, pandemic context	High
Miller DJ et al., 2022[98]	Australia	Cross-sectional	53 healthy adults (27 men, 25.4 ± 5.9 years)	All of Oura, Apple watch, Polar, Garmin, Somfit	Multi-device sleep/heart rate/heart rate variability validation	Sleep: 89% agreement; HR: r=0.85; HRV: r=0.63 (vs polysomnography)	Single night, healthy participants only	Low

Niela-Vilen H et al., 2022[99]	Finland	Cross-sectional	42 healthy adults (median age 32 years, range, 18–46 years)	Oura	Validate steps and sedentary time vs ActiGraph	Strong correlation steps (r=0.83) but overestimated by 1416 steps; sedentary time overestimated 17 min	Small convenience sample, 1-week measurement only	Moderate
Rovini E et al., 2021[100]	Italy	Case-control	10 Healthy controls (6 women, 63.7 ± 9.9 years) vs 8 mild cognitive impairment patients (7 women, 75.7 ± 5.2 years)	SensRing	Detect action planning impairments	Successfully differentiated healthy controls from mild cognitive impairment patients	Custom device, small sampl	Low
Ahn C et al., 2021[101]	Korea	Randomized crossover	20 healthy volunteers (15 men, median 23 years for men and 21 years for women)	Self-developed smart ring	Improve chest compression quality during cardiopulmonary resuscitation with the aid of smart ring	Accurate-depth chest compression proportion 88.7% vs 22.6% (p=0.033)	Small sample, simulated environment, limited generalizability	Moderate
Henriksen A et al., 2021[102]	Norway	Observational	113 participants (59 women, 40.6 ± 10.6 years)	Oura	Physical activity monitoring system development	System successfully collected multi-provider data	No participants used Oura in final analysis	Moderate
Jimah T et al., 2021[103]	US	Case report	1 Pregnant woman (age 29)	Oura	Technology-based pregnancy health intervention	Correlations: positive emotions-heart rate variability (r=0.54), sleep quality-score (r=0.52)	Case report, not generalizable	High
Lee S et al., 2021[104]	Korea	Proof-of-concept	4 emergency medical professionals	Self-developed smart ring	Chest compression depth estimation	Absolute errors: 2.0 ± 1.1mm, 2.2 ± 0.9mm, 1.4 ± 1.1mm across situations	Small validation sample, prototype device	High
Moshe I et al., 2021[105]	Finland (international recruit)	Observational	55 adults (30 women, 42.8 ± 11.6 years)	Oura and iphone	Predict depression/anxiety symptoms during COVID-19 pandemic	A combined model of smartphone and wearable features and self-reported mood provided the strongest prediction of depression	Small sample, COVID-19 context, non-clinical population	Moderate
Kinnunen H et al., 2020[106]	Finland	Cross-sectional	49 healthy adults (31.6 ± 11.8 years)	Oura	Validate nocturnal heart rate and heart rate variability	high agreement: heart rate r^2=0.996, heart rate variability r^2=0.980	Healthy participants only, nocturnal measurements only	Low

					accuracy vs electrocardiography			
Maijala A et al., 2019[107]	Finland	Pilot study	22 volunteer women (34.7 ± 8.8 years),	Oura	Menstrual cycle tracking via temperature	Temperature differences 0.30°C skin vs 0.23°C oral; menstruation sensitivity 71.9-86.5%	Small sample, pilot study, non-calibrated sensors	Moderat e

AUC, area under the curve

Supplementary Table S3. Risk of bias evaluation (ROBINS-I assessment tool) for sleep studies

Study	Bias pre-intervention and at intervention domains			Risk of bias post-intervention domains				Overall assessment of bias
	Bias due to confounding	Bias in selection of participants into the study	Bias in classification of intervention	Bias due to deviations from intended intervention	Bias due to missing data	Bias in measurement of outcomes	Bias in selection of the reported result	
Bigalke JA et al., 2025[1]	Low	Low	Low	Low	Low	Moderate (No polysomnography data)	Low	Moderate
Ng ASC et al., 2025[2]	Low	Moderate (healthy university students only	Low	Low	Low	Low	Low	Moderate
Meira E Cruz M et al., 2025[3]	Low	Low	Low	Low	Low	Low	Low	Low
Herberger S et al., 2025[4]	Moderate (patient heterogeneity	Moderate (clinical population only)	Low	Low	Low	Moderate (single night)	Low	Moderate
Ju E et al., 2025[5]	Moderate (unmeasured confounders)	Low	Low	Low	Low	Moderate (No polysomnography data)	Low	Moderate
Barba E et al., 2025[6]	Moderate (no training load considered)	Low	Low	Low	Moderate (adherence not reported)	Moderate (sleep staging uncertainty)	Low	Moderate

Willoughby AR et al., 2025[7]	Low	Low	Low	Low	Low	Low	Low	Low
Montesino-Goicolea S et al., 2025[8]	Serious (secondary analysis)	Moderate (patents with chronic pain only)	Low	Low	Low	Moderate (No polysomnograph y data, self-report reference)	Moderate (post-hoc)	High
Vatne E et al., 2025[9]	Low	Moderate (athletes only)	Low	Low	Low	Moderate (self-selection)	Low	Moderate
Qin S et al., 2025[11]	Moderate (survivorship bias)	Moderate (age limited)	Low	Low	Low	Moderate (no obstructive sleep apnea assessment	Low	Moderate
Soon CS et al., 2025[12]	Low	Moderate (single university setting, freshmen only)	Low	Low	Serious (decreasing participation)	Moderate (No polysomnograph y data)	Low	High
Moen LV et al., 2025[14]	Low	Moderate (work restriction)	Low	Low	Low	Moderate (No polysomnograph y data)	Low	Moderate
Singh A et al., 2025[15]	Serious (only cases)	Serious (only cases)	Low	Low	Low	Serious (data quality varies, No polysomnograph y data)	Low	High
Font R et al., 2025[16]	Moderate (single team)	Moderate (handball players)	Low	Low	Moderate (match restrictions)	Moderate (No polysomnograph y data)	Low	Moderate

Debbiche I et al. 2025[17]	Serious (secondary analysis)	Low	Low	Low	Low	Moderate (device limitations for passive wake detection)	Low	High
Donahue CC et al. 2025[18]	Moderate (no pre-injury baseline data)	Moderate (small matched sample)	Low	Low	Low	Moderate (No polysomnograph y data)	Low	Moderate
Massar SAA et al., 2024[20]	Low	Moderate (hospital setting)	Low	Low	Low	Moderate (No polysomnograph y data)	Low	Moderate
Robbins R et al., 2024[21]	Low	Low	Low	Low	Low	Low	Low	Low
Labbaf S et al., 2024[22]	Moderate (COVID context)	Moderate (college setting)	Low	Low	Low	Moderate (No polysomnograph y data)	Low	Moderate
Viswanath VK et al., 2024[23]	Low	Low	Low	Low	Low	Moderate (self-report reference)	Low	Moderate
Fudolig MI et al., 2024[24]	Low	Moderate (university setting)	Low	Low	Low	Moderate (self-report mental health)	Low	Moderate
Willoughby AR et al., 2024[28]	Moderate (laboratory setting)	Low	Low	Low	Low	Low	Low	Moderate
Svensson T et al., 2024[29]	Moderate (hotel setting)	Low	Low	Low	Low	Low	Low	Moderate
Kainec KA et al., 2024[30]	Low	Low	Low	Low	Low	Moderate (single night)	Low	Moderate

Kubala AG et al., 2024[31]	Low	Moderate (shipboard setting)	Low	Low	Moderate (variable adherence)	Moderate (operational environment)	Low	Moderate
Ong JL et al., 2024[32]	Low	Low	Low	Low	Low	Low	Low	Low
Hall LS et al., 2024[33]	Serious (occupational stress)	Moderate (farmers only)	Low	Low	Serious (57% wear time)	Moderate (No polysomnograph y data)	Low	High
Ou YH et al., 2023[34]	Moderate (Hypertensive patients	Low	Low	Low	Low	Low	Low	Moderate
Lee T et al., 2023[35]	Moderate (single night, laboratory setting)	Low	Low	Low	Low	Low	Low	Moderate
Nauha L et al., 2023[37]	Low	Low	Low	Low	Low	Moderate (self-report reference)	Low	Moderate
Chinoy ED et al., 2023[38]	Low	Low	Low	Low	Low	Moderate (self-report reference)	Low	Moderate
Shuster AE et al., 2023[39]	Low	Low	Low	Low	Low	Moderate (self-report mood)	Low	Moderate
de Vries HJ et al., 2023[40]	Moderate (COVID context)	Serious (n=8)	Low	Low	Moderate (variable adherence)	Moderate (subjective stress)	Low	High
Grandner MA et al., 2023[41]	Low	Low	Low	Low	Low	Low	Low	Low

Chinoy ED et al., 2022[42]	Low	Low	Low	Low	Low	Moderate (daytime sleep detection)	Low	Moderate
Zhao R et al., 2022[43]	Low	Low	Low	Low	Low	Low	Low	Low
Mousavi ZA et al., 2022[44]	Moderate (COVID context)	Moderate (college students only)	Low	Low	Low	Moderate (self-report affect)	Low	Moderate
Rentz LE et al., 2022[45]	Low	Moderate (athletes only)	Low	Low	Low	Moderate (No polysomnography data)	Low	Moderate
Ghorbani S et al., 2022[46]	Moderate (healthy only)	Low	Low	Low	Low	Low	Low	Moderate
Henriksen A et al., 2022[47]	Low	Low	Low	Low	Low	Moderate (diary comparison)	Low	Moderate
Roberts DM et al., 2020[48]	Moderate (laboratory setting)	Serious (n=8)	Low	Low	Low	Moderate (No polysomnography data)	Low	High
Stone JD et al., 2020[49]	Moderate (demographics)	Serious (n=5)	Low	Low	Low	Moderate (No polysomnography data)	Low	High
Chaudhry FF et al., 2020[50]	Serious (multiple confounders)	Low	Low	Low	Serious	Moderate (No polysomnography data, self reporting)	Moderate (selective reporting)	High
Asgari Mehrabadi M et al. 2020[51]	Low	Low	Low	Low	Low	Moderate (No polysomnography data)	Low	Moderate

Supplementary Table S4. Risk of bias 2.0 (ROB 2.0 tool) for sleep studies

**Study/year**	**Randomization process**	**Deviation from the intervention**	**Missing outcome data**	**Measurement of the outcome**	**Selection of the reported result**	**Overall**
Norful AA et al., 2025[10]	High (single arm)	Some concerns (no control)	Some concerns (25% dropout)	Some concerns (no polysomnography data)	Low	High
Smith ES et al., 2025[13]	Low	Low	Low	Some concerns (secondary outcomes)	Low	Moderate
Fein T et al. 2025[19]	High (randomization method not clearly described)	Low	High (only 8/15 achieved target dehydration)	Some concerns (no polysomnography data)	Low	High
Armitage BT et al., 2024[25]	Low	High risk (COVID disruption)	High risk (COVID disruption)	Some concerns (self-report)	Low	High
Hausenblas HA et al., 2024[26]	Low	Low	Low	Low	Low	Low
Breus M et al., 2024[27]	High (no randomization)	Some concerns (no bliding)	Low	Some concerns (subjective measurement)	Some concerns (no protocol)	High
Vanhanen J et al., 2023[36]	Low	Low	Low	Some concerns (no polysomnography data)	Low	Moderate

Supplementary Table S5. Risk of bias evaluation (ROBINS-I assessment tool) for non-sleep studies

Study	Bias pre-intervention and at intervention domains			Risk of bias post-intervention domains				Overall assessment of bias
	Bias due to confounding	Bias in selection of participants into the study	Bias in classification of intervention	Bias due to deviations from intended intervention	Bias due to missing data	Bias in measurement of outcomes	Bias in selection of the reported result	
Borelli JL et al., 2025[52]	Moderate (undergraduate only)	Moderate (specific age group)	Low	Low	Moderate (28% dropout)	Low	Low	Moderate
Dupuit M et al., 2025[53]	Serious (n=2, case report)	Serious (n=2, case report)	Low	Low	Low	Low	Low	High
Hadid A et al., 2025[54]	Low	Moderate (healthy adults only)	Low	Low	Low	Low	Low	Moderate
Hanish RR et al., 2025[55]	Moderate (obsessive-compulsive disorder specific)	Low	Low	Low	Moderate (subset analysis)	Low	Low	Moderate
Hirten IP et al., 2025[56]	Moderate (disease-specific)	Low	Low	Low	Low	Low	Low	Moderate
Ortiz A et al., 2025[57]	Moderate (disease-specific)	Moderate (disease-specific)	Low	Low	Low	Low	Low	Moderate
Reithe H et al., 2025[58]	Moderate (disease-specific)	Moderate (disease-specific)	Low	Low	Low	Moderate (device limitations)	Low	Moderate

Rim J et al., 2025[59]	Serious (n=9)	Low	Low	Low	Low	Low	Low	High
Thigpen N et al., 2025[60]	Low	Low	Low	Low	Low	Low	Low	Low
van Steijn NJ et al., 2025[61]	Low	Low	Low	Low	Low	Low	Low	Low
Zakaria H et al., 2025[62]	Critical (n=1)	Critical (single case)	Low	Low	Low	Moderate (individual response	Moderate (case-specific)	High
Morimoto M et al., 2024[63]	Low	Moderate (surgery patients only)	Low	Low	Low	Low	Low	Moderate
Mastrototaro JJ et al., 2024[64]	Low	Moderate (healthy volunteers only)	Low	Low	Low	Low	Low	Moderate
Mason et AE al., 2024[65]	Low	Moderate (depression subset)	Low	Low	Low	Moderate (self-reported outcomes)	Low	Moderate
Liang T et al., 2024[66]	Low	Low	Low	Low	Low	Low	Low	Low
Kim J et al., 2024[67]	Low	Low	Low	Low	Low	Low	Low	Low
Keeler Bruce L et al., 2024[68]	Low	Low	Low	Low	Low	Low	Low	Low
Kasl P et al., 2024[69]	Moderate (limited validation, only population level)	Low	Low	Low	Low	Low	Low	Moderate

Ibrahim AH et al., 2024[70]	Moderate (exercisers only)	Moderate (exercisers only)	Low	Low	Low	Low	Low	Moderate
D'Adamo CR et al., 2024[71]	Low	Low	Low	Moderate (device variable use)	Low	Moderate (subjective measures)	Low	Moderate
Alzueta E et al., 2024[72]	Moderate (healthy subjects only)	Low	Low	Low	Low	Moderate (single cycle)	Low	Moderate
Basavaraj C et al., 2024[73]	Moderate (healthy pregnant women only	Moderate (specific population)	Low	Low	Low	Moderate (proprietary metrics)	Low	Moderate
Peterson NE et al., 2024[74]	Low	Moderate (women only	Low	Low	Low	Moderate (subjective motivation)	Low	Moderate
Phipps J et al., 2024[75]	Low	Moderate (self-selected)	Low	Low	Low	Moderate (self-reported)	Low	Moderate
Ryan JM et al., 2024[76]	Moderate (Only COVID patients)	Low	Low	Low	Low	Low	Low	Moderate
Sharifi-Heris Z et al., 2024[77]	Moderate (Hispanic only)	Low	Low	Low	Low	Low	Low	Moderate
Kristiansson E et al., 2023[79]	Low	Moderate (ages restricted)	Low	Low	Low	Low	Low	Moderate
Björkman K et al., 2023[80]	Low	Low	Low	Low	Low	Low	Low	Low
Balsam D et al., 2023[81]	Moderate (no control group)	Serious (self-selection bias)	Low	Moderate (app adherence variable)	Low	Moderate (self-reported outcomes)	Low	High

Bruce LK et al., 2023[82]	Low	Low	Low	Low	Low	Moderate (single device)	Low	Moderate
Croghan IT et al., 2023[83]	Moderate (no control group)	Serious (n=10)	Low	Low	Low	Low	Low	High
Erickson EN et al., 2023[84]	Moderate (healthy pregnant group)	Low	Low	Low	Low	Low	Low	Moderate
Jafarlou S et al., 2023[85]	Moderate (COVID impact)	Serious (only 7/20 complete data)	Low	Low	Serious (only 7/20 complete data)	Low	Low	High
Nolasco HR et al., 2023[86]	Moderate (unspecified population)	Moderate (unspecified population)	Low	Low	Low	Low	Low	Moderate
Padmanabha A et al., 2023[87]	Moderate (prototype)	Moderate (unspecified population)	Low	Low	Low	Low	Low	Moderate
Sanai F et al., 2023[88]	Moderate (prototype)	Moderate (prototype)	Low	Low	Low	Moderate (accuracy variation)	Low	Moderate
Shiba SK et al., 2023[89]	Moderate (healthcare workers only, early pandemic	Moderate (healthcare workers only, early pandemic	Low	Low	Low	Low	Low	Moderate
Mason AE et al., 2022[90]	Moderate (73 positive only)	Moderate (self-selected)	Low	Low	Low	Low	Low	Moderate
Mason AE et al., 2022[91]	Low	Low	Low	Low	Low	Low	Low	Low
Alzueta E et al., 2022[92]	Moderate (no control group,	Low	Low	Low	Low	Moderate (single cycle)	Low	Moderate

	healthy subjects only)							
Cao R et al., 2022[93]	Low	Low	Low	Low	Low	Low	Low	Low
Conroy B et al., 2022[94]	Moderate (symptomatic only)	Moderate (48% data capture needed)	Low	Low	Low	Low	Low	Moderate
de Vries H et al., 2022[95]	Moderate (occupation-specific)	Moderate (occupation-specific)	Low	Low	Low	Low	Low	Moderate
Fonseka RD et al., 2022[96]	Serious (n=1, case report)	Serious (n=1, case report)	Low	Low	Low	Low	Low	High
Jimah T et al., 2022[97]	Serious (n=2, case report)	Serious (n=2, case report)	Low	Low	Low	Low	Low	High
Miller DJ et al., 2022[98]	Low	Low	Low	Low	Low	Low	Low	Low
Niela-Vilen H et al., 2022[99]	Low	Moderate (convenience sample)	Low	Low	Low	Low	Low	Moderate
Rovini E et al., 2021[100]	Low	Low	Low	Low	Low	Low	Low	Low
Henriksen A et al., 2021[102]	Moderate (no final validation)	Moderate (system development)	Low	Low	Moderate (no final validation)	Moderate (indirect measurement)	Low	Moderate
Jimah T et al., 2021[103]	Serious (n=1, case report)	Serious (n=1, case report)	Low	Low	Low	Moderate (self-reported outcomes)	Low	High

Lee S et al., 2021[104]	Moderate (prototype)	Serious (n=4 validation)	Low	Low	Low	Low	Low	High
Moshe I et al., 2021[105]	Moderate (COVID context)	Low	Low	Low	Low	Low	Low	Moderate
Kinnunen H et al., 2020[106]	Low	Low	Low	Low	Low	Low	Low	Low
Maijala A et al., 2019[107]	Moderate (pilot study)	Low	Low	Low	Moderate (sensor calibration)	Low	Low	Moderate

Supplementary Table S6. Risk of bias 2.0 (ROB 2.0 tool) for non-sleep studies

Study/year	Randomization process	Deviation from the intervention	Missing outcome data	Measurement of the outcome	Selection of the reported result	Overall
Liew SJ et al., 2023[78]	Low	Some concerns (non-blinded)	Low	Some concerns (outcome assessors not blinded, mix of objective and subjective measures)	Low	Moderate
Ahn C et al., 2021[101]	Low	Some concerns (simulated environment, not real cardiopulmonary resuscitation)	Low	Low	Low	Moderate

References

1. Bigalke JA, Lee KM, Bigalke JR, et al. Longitudinal assessment of objective sleep and power output in Division I collegiate baseball athletes. Sleep Health 2025;11(2):241-247. doi: 10.1016/j.sleh.2024.12.009.
2. Ng ASC, Shyong Tai E, Chee MWL. Effects of night-to-night variations in objectively measured sleep on blood glucose in healthy university students. Sleep. 2025;48(2):zsae224. doi: 10.1093/sleep/zsae224.
3. Meira e Cruz M, Chen E, Zhou Y, et al. A wearable ring oximeter for detection of sleep disordered breathing. Respir Med. 2025:242:108092. doi: 10.1016/j.rmed.2025.108092.
4. Herberger S, Aurnhammer C, Bauerfeind S, et al. Performance of wearable finger ring trackers for diagnostic sleep measurement in the clinical context. Sci Rep. 2025;15(1):9461. doi: 10.1038/s41598-025-93774-z.
5. Ju E, Guo Y, Park JI, et al. Sleep Quality of Persons with Dementia and Family Caregivers in Korean Americans: Wearable Technology to Study the Dyadic Association. West J Nurs Res. 2025;47(5):404-412. doi: 10.1177/01939459251323675. Epub 2025 Mar 17.
6. Barba E, Casamichana D, Figueiredo P, Nakamura FY, Castellano J. The Use of Principal Component Analysis for Reduction in Sleep Quality and Quantity Data in Female Professional Soccer. Sensors (Basel). 2024 Dec 30;25(1):148. doi: 10.3390/s25010148.

7. Willoughby AR, Vallat R, Ong JL, Chee MWL. Insights about travel-related sleep disruption from 1.5 million nights of data. Sleep. 2025;48(7):zsaf077. doi: 10.1093/sleep/zsaf077.

8. Montesino-Goicolea S, Valdes-Hernandez PA, Nin O, Smith C, Porges EC, Cruz-Almeida Y. Assessing Self-reported and Device-Derived Sleep Quality in a Sample of Older Adults with Chronic Musculoskeletal Pain. medRxiv [Preprint]. 2025:2025.03.17.25324131. doi: 10.1101/2025.03.17.25324131.

9. Vatne E, Merrigan JJ, Stone JD, et al. Effects of Daytime Floatation-Restricted Environmental Stimulation Therapy on Nocturnal Cardiovascular Physiology, Sleep, and Subjective Recovery in Collegiate Student-Athletes: A Comprehensive Observational Study. J Strength Cond Res. 2025;39(8):857-867. doi: 10.1519/JSC.0000000000005131.

10. Norful AA, de Jacq K, Zhao J, et al. Exploring longitudinal physiologic stress measurement and sleep quality interventions to improve psychological well-being in nurses: a pilot study. Health Psychol Behav Med. 2025;13(1):2503376. doi: 10.1080/21642850.2025.2503376. eCollection 2025.

11. Qin S, Ng EKK, Soon CS, et al. Association between objectively measured, multidimensional sleep health and cognitive function in older adults: cross-sectional wearable tracker study. Sleep Med. 2025;132:106569. doi: 10.1016/j.sleep.2025.106569.

12. Soon CS, Chua XY, Leong RLF, et al. A Longitudinal Study of Sleep in University Freshmen: Facilitating and Impeding Factors. Sleep. 2025:zsaf156. doi: 10.1093/sleep/zsaf156

13. Smith ES, Kuikman M, Rusell S, et al. 24-Hour Low Energy Availability Induced by Diet or Exercise Exhibits Divergent Influences on Sleep and Recovery Indices among Female and Male Cyclists. Med Sci Sports Exerc. 2025. Online ahead of print. doi: 10.1249/MSS.0000000000003783.

14. Moen LV, Rysstad TL, Lie JS, Haugen F, Matre D. Validation of the Norwegian version of the Munich ChronoType Questionnaire for shift workers ($MCTQ^{Shift}$). Chronobiol Int. 2025;42(8):1013-1024. doi: 10.1080/07420528.2025.2523514.

15. Singh A, Cai Y, Mason AE, Chang JL. Changes in Wearable Sleep Technology Metrics During Hypoglossal Nerve Stimulation Uptitration. Laryngoscope. 2025. Online ahead of print. doi: 10.1002/lary.32465.

16. Font R, Ortega-Becerra M, Tremps V, et al. Analysis of sleep quality and quantity during a half-season in world-class handball players. Biol Sport. 2025;42(3):247-255. doi: 10.5114/biolsport.2025.148578.

17. Debbiche I, Wang CC, Gomez-Roas M, et al. Rest assured: High sleep efficiency reduces postoperative complications and opioid prescriptions in patients undergoing surgeries with gynecologic oncologists. Gynecol Oncol. 2025:194:119-124. doi: 10.1016/j.ygyno.2025.01.007.

18. Donahue CC, Barnes LE, Hertel JN, Resch JE. Acute Changes in Sleep Stages Following Concussion in Collegiate Athletes: A Pilot Study. J Athl Train. 2025. doi: 10.4085/1062-6050-0629.24. Online ahead of print.

19. Fein T, Garay J, Voss MA. Effects of Fluid Intake on Sleep Duration and Quality Among Healthy Adults. Nat Sci Sleep. 2025:17:791-800. doi: 10.2147/NSS.S511807. eCollection 2025.

20. Massar SAA, Chua XY, Leong R, et al. Sleep, Well-Being, and Cognition in Medical Interns on a Float or Overnight Call Schedule. JAMA Netw Open. 2024;7(10):e2438350. doi: 10.1001/jamanetworkopen.2024.38350.

21. Robbins R, Weaver MD, Sullivan JP, et al. Accuracy of Three Commercial Wearable Devices for Sleep Tracking in Healthy Adults. Sensors (Basel). 2024;24(20):6532. doi: 10.3390/s24206532.

22. Labbaf S, Abbasian M, Nguyen B, et al. Physiological and emotional assessment of college students using wearable and mobile devices during the 2020 COVID-19 lockdown: An intensive, longitudinal dataset. Data Brief. 2024:54:110228. doi: 10.1016/j.dib.2024.110228. eCollection 2024 Jun.

23. Viswanath VK, Hartogenesis W, Dilchert S, et al. Five million nights: temporal dynamics in human sleep phenotypes. NPJ Digit Med. 2024;7(1):150. doi: 10.1038/s41746-024-01125-5.

24. Fudolig MI, Bloomfield LSP, Price M, et al. The Two Fundamental Shapes of Sleep Heart Rate Dynamics and Their Connection to Mental Health in College Students. Digit Biomark. 2024;8(1):120-131. doi: 10.1159/000539487.

25. Armitage BT, Potts HWW, Irwin MR, Fisher A. Exploring the Impact of a Sleep App on Sleep Quality in a General Population Sample: pilot Randomized Controlled Trial. JMIR Form Res. 2024:8:e39554. doi: 10.2196/39554.

26. Hausenblas HA, Lynch T, Hooper S, et al. Magnesium-L-threonate improves sleep quality and daytime functioning in adults with self-reported sleep problems: a randomized controlled trial. Sleep Med X. 2024:8:100121. doi: 10.1016/j.sleepx.2024.100121

27. Breus M, Hooper SL, Lynch T, et al. Effectiveness of a grid mattress on adults' sleep quality and health: A quasi-experimental intervention study. Health Sci Rep. 2024;7(4):e2046. doi: 10.1002/hsr2.2046.

28. Willoughby AR, Golkashani HA, Ghorbani S, et al. Performance of wearable sleep trackers during nocturnal sleep and periods of simulated real-world smartphone use. Sleep Health. 2024;10(3):356-368. doi: 10.1016/j.sleh.2024.02.007.

29. Svensson T, Madhawa K, Nt H, et al. Validity and reliability of the Oura Ring Generation 3 (Gen3) with Oura sleep staging algorithm 2.0 (OSSA 2.0) when compared to multi-night ambulatory polysomnography: A validation study of 96 participants and 421,045 epochs. Sleep Medicine. 2024;115:251-263. doi: 10.1016/j.sleep.2024.01.020. Epub 2024 Jan 26.

30. Kainec KA, Caccavaro J, Barnes M, et al. Evaluating Accuracy in Five Commercial Sleep-Tracking Devices Compared to Research-Grade Actigraphy and Polysomnography. Sensors (Basel). 2024;24(2):635. doi: 10.3390/s24020635.

31. Kubala AG, Roma PG, Jameson JT, et al. Advancing a U.S. navy shipboard infrastructure for sleep monitoring with wearable technology. Appl Ergon. 2024:117:104225. doi: 10.1016/j.apergo.2024.104225.

32. Ong JL, Golkashani HA, Ghorbani S, et al. Selecting a sleep tracker from EEG-based, iteratively improved, low-cost multisensor, and actigraphy- only devices. Sleep Health. 2024;10(1):9-23. doi: 10.1016/j.sleh.2023.11.005.

33. Hall LS, Edwards JP, Dale K, et al. An exploration into the sleep of workers on block-calving, pasture-based dairy farms. J Dairy Sci. 2024;107(11):9549-9557. doi: 10.3168/jds.2024-24969.

34. Ou YH, Ong J, Thant AT, et al. The Belun sleep platform to diagnose obstructive sleep apnea in patients with hypertension and high cardiovascular risk. J Hypertens. 2023;41(6):1011-1017. doi: 10.1097/HJH.0000000000003426.

35. Lee T, Cho Y, Cha KS, et al. Accuracy of 11 Wearable, Nearable, and Airable Consumer Sleep Trackers: Prospective Multicenter Validation Study. JMIR Mhealth Uhealth. 2023:11:e50983. doi: 10.2196/50983.

36. Vanhanen J, Kujala J, Liljeström M, et al. rTMS targeted to the secondary somatosensory cortex influences sleep in CRPS patients, as measured with the OURA ring. Brain Behav. 2023;13(11):e3252. doi: 10.1002/brb3.3252.

37. Nauha L, Farrahi V, Jurvelin H, et al. Comparison and agreement between device-estimated and self-reported sleep periods in adults. Ann Med. 2023;55(1):2191001. doi: 10.1080/07853890.2023.2191001.

38. Chinoy ED, Cuellar JA, Jameson JT, Markwald RR. Daytime Sleep-Tracking Performance of Four Commercial Wearable Devices During Unrestricted Home Sleep. Nat Sci Sleep. 2023;15:151-164. doi: 10.2147/NSS.S395732. eCollection 2023.

39. Shuster AE, Simon KC, Zhang J, et al. Good sleep is a mood buffer for young women during menses. Sleep. 2023;46(10):zsad072. doi: 10.1093/sleep/zsad072.

40. de Vries HJ, Pennings HJM, van der Schans CP, et al. Wearable-Measured Sleep and Resting Heart Rate Variability as an Outcome of and Predictor for Subjective Stress Measures: A Multiple N-of-1 Observational Study. Sensors (Basel). 2022;23(1):332. doi: 10.3390/s23010332.

41. Grandner MA, Bromberg Z, Hadley A, et al. Performance of a multisensor smart ring to evaluate sleep: in-lab and home-based evaluation of generalized and personalized algorithms. Sleep. 2023;46(1):zsac152. doi: 10.1093/sleep/zsac152.

42. Chinoy ED, Cuellar JA, Jameson JT, Markwald RR. Performance of Four Commercial Wearable Sleep-Tracking Devices Tested Under Unrestricted Conditions at Home in Healthy Young Adults. Nat Sci Sleep. 2022;14:493-516. doi: 10.2147/NSS.S348795. eCollection 2022.

43. Zhao R XJ, Zhang X, Peng M, et al. Comparison of Ring Pulse Oximetry Using Reflective Photoplethysmography and PSG in the Detection of OSA in Chinese Adults: A Pilot Study. Nat Sci Sleep. 2022;14:1427-1436. doi: 10.2147/NSS.S367400. eCollection 2022.

44. Mousavi ZA, Lai J, Simon K, et al. Sleep Patterns and Affect Dynamics Among College Students During the COVID-19 Pandemic: Intensive Longitudinal Study. JMIR Form Res. 2022;6(8):e33964. doi: 10.2196/33964.

45. Rentz LE, Bryner RW, Ramadan J, Rezai A, Galster SM. Full-Body Photobiomodulation Therapy Is Associated with Reduced Sleep Durations and Augmented Cardiorespiratory Indicators of Recovery. Sports (Basel). 2022;10(8):119. doi: 10.3390/sports10080119.

46. Ghorbani S, Golkashani HA, Chee NIYN, et al. Multi-Night at-Home Evaluation of Improved Sleep Detection and Classification with a Memory- Enhanced Consumer Sleep Tracker. Nat Sci Sleep. 2022;14:645-660. doi: 10.2147/NSS.S359789. eCollection 2022.

47. Henriksen A, Svartdal F, Grimsgaard S, Hartvigsen G, Hopstock LA. Polar Vantage and Oura Physical Activity and Sleep Trackers: Validation and Comparison Study. JMIR Form Res. 2022;6(5):e27248. doi: 10.2196/27248.

48. Roberts DM, Schade MM, Mathew GM, Gartenberg D, Buxton OM. Detecting sleep using heart rate and motion data from multisensor consumer-grade wearables, relative to wrist actigraphy and polysomnography. Sleep 2020;43(7):1-19. doi: 10.1093/sleep/zsaa045.

49. Stone JD, Rentz LE, Forsey J, et al. Evaluations of Commercial Sleep Technologies for Objective Monitoring During Routine Sleeping Conditions. Nat Sci Sleep. 2020;12:821-842. doi: 10.2147/NSS.S270705. eCollection 2020.

50. Chaudhry FF, Danieletto M, Golden E, et al. Sleep in the Natural Environment: A Pilot Study. Sensors (Basel). 2020;20(5):1378. doi: 10.3390/s20051378.

51. Asgari Mehrabadi M, Azimi I, Sarhaddi F, et al. Sleep Tracking of a Commercially Available Smart Ring and Smartwatch Against Medical- Grade Actigraphy in Everyday Settings: Instrument Validation Study. JMIR Mhealth Uhealth. 2020;8(10):e20465. doi: 10.2196/20465.

52. Borelli JL, Wang Y, Li FH, et al. Detection of Depressive Symptoms in College Students Using Multimodal Passive Sensing Data and Light Gradient Boosting Machine: Longitudinal Pilot Study. JMIR Form Res. 2025:9:e67964. doi: 10.2196/67964.

53. Dupuit M, Barlier K, Tranchard B, et al. Heart Rate Variability Measurements Across the Menstrual Cycle and Oral Contraceptive Phases in Two Olympian Female Swimmers: A Case Report. Sports (Basel). 2025;13(6):185. doi: 10.3390/sports13060185.

54. Hadid A, McDonald EG, Ding Q, et al. Development of machine learning prediction models for systemic inflammatory response following controlled exposure to a live attenuated influenza vaccine in healthy adults using multimodal wearable biosensors in Canada: a single-centre, prospective controlled trial. Lancet Digit Health. 2025;7(7):100886. doi: 10.1016/j.landig.2025.100886.

55. Hanish RR, Kutcher TP, Frączek TM, et al. Artifact Identification and Mitigation Strategies for Longitudinal Neural Data Collection Onboard the Medtronic Percept DBS Device. medRxiv[Preprint]. 2025:2025.07.23.25331987. doi: 10.1101/2025.07.23.25331987.

56. Hirten RP, Danieletto M, Sanchez-Mayor M, et al. Physiological Data Collected From Wearable Devices Identify and Predict Inflammatory Bowel Disease Flares. Gastroenterology. 2025;168(5):939-951.e5. doi: 10.1053/j.gastro.2024.12.024.

57. Ortiz A, Halabi R, Alda M, et al. Day-to-day variability in activity levels detects transitions to depressive symptoms in bipolar disorder earlier than changes in sleep and mood. Int J Bipolar Disord. 2025;13(1):13. doi: 10.1186/s40345-025-00379-6.

58. Reithe H, Marty B, Torrado JC, et al. Cross-evaluation of wearable data for use in Parkinson's disease research: a free-living observational study on Empatica E4, Fitbit Sense, and Oura. Biomed Eng Online. 2025;24(1):22. doi: 10.1186/s12938-025-01353-0.

59. Rim J, Xu Q, Tang X, Jimah T, Guo Y, Qu A. Heterogeneous effects of physical activity on physiological stress during pregnancy. medRxiv [Preprint]. 2025 Mar 31:2025.03.30.25324909. doi: 10.1101/2025.03.30.25324909.

60. Thigpen N, Patel S, Zhang X. Oura Ring as a Tool for Ovulation Detection: Validation Analysis. J Med Internet Res. 2025:27:e60667. doi: 10.2196/60667.

61. van Steijn NJ, Pepplinkhuizen S, Postema PG, et al. Ventricular arrhythmia detection with a wearable ring-type photoplethysmography sensor: A feasibility study. Heart Rhythm. 2025:S1547-5271(25)02554-8. doi: 10.1016/j.hrthm.2025.06.008.

62. Zakaria H, Roman I, Almarzooqi I, Hashemi A. Integrating Wearables Into a Hybrid Care Model: Continuous Remote Monitoring of a Diabetic Patient With Frequent Premature Ventricular Contractions Using an ŌURA Ring. Cureus. 2025;17(6):e86174. doi: 10.7759/cureus.86174. eCollection 2025 Jun.

63. Morimoto M, Nawari A, Savic R, Marmor M. Exploring the Potential of a Smart Ring to Predict Postoperative Pain Outcomes in Orthopedic Surgery Patients. Sensors (Basel). 2024;24(15):5024. doi: 10.3390/s24155024.

64. Mastrototaro JJ, Leabman M, Shumate J, Tompkins KL. Performance of a Wearable Ring in Controlled Hypoxia: A Prospective Observational Study. JMIR Form Res. 2024;8:e54256. doi: 10.2196/54256.

65. Mason AE, Kasl P, Soltani S, et al. Elevated body temperature is associated with depressive symptoms: results from the TemPredict Study. Sci Rep. 2024;14(1):1884. doi: 10.1038/s41598-024-51567-w.

66. Liang T, Yilmaz G, Soon CS. Deriving Accurate Nocturnal Heart Rate, rMSSD and Frequency HRV from the Oura Ring. Sensors (Basel). 2024;24(23):7475. doi: 10.3390/s24237475.

67. Kim J, Chang SA, Park SW. First-in-Human Study for Evaluating the Accuracy of Smart Ring Based Cuffless Blood Pressure Measurement. J Korean Med Sci. 2024;39(2):e18. doi: 10.3346/jkms.2024.39.e18.

68. Keeler Bruce L, González D, Dasgupta S, Smarr BL. Biometrics of complete human pregnancy recorded by wearable devices NPJ Digit Med. 2024;7(1):207. doi: 10.1038/s41746-024-01183-9.

69. Kasl P, Keeler Bruce L, Hartogensis W, et al. Utilizing Wearable Device Data for Syndromic Surveillance: A Fever Detection Approach. Sensors (Basel). 2024;24(6):1818. doi: 10.3390/s24061818.

70. Ibrahim AH, Beaumont CT, Strohacker K. Exploring Regular Exercisers' Experiences with Readiness/Recovery Scores Produced by Wearable Devices: A Descriptive Qualitative Study. Appl Psychophysiol Biofeedback. 2024 Sep;49(3):395-405. doi: 10.1007/s10484-024-09645-2.

71. D'Adamo CR, Denmark D, Carreras-Gallo N, et al. A Pilot Study Evaluating the Feasibility and Efficacy of an In-Home Resonance-Based Electromagnetic Field Protection Device on Improving Markers of Health and Cognitive Function Among a Sample of Healthy Adults. Integr Med (Encinitas). 2024;23(4):23-28.

72. Alzueta E, Gombert-Labedens M, Javitz H, et al. Menstrual Cycle Variations in Wearable-Detected Finger Temperature and Heart Rate, But Not in Sleep Metrics, in Young and Midlife Individuals. J Biol Rhythms. 2024;39(5):395-412. doi: 10.1177/07487304241265018.

73. Basavaraj C, Grant AD, Aras SG, Erickson EN. Deep learning model using continuous skin temperature data predicts labor onset. BMC Pregnancy Childbirth. 2024;24(1):777. doi: 10.1186/s12884-024-06862-9.

74. Peterson NE, Bate DA, Macintosh JL, Trujillo Tanner C. Wearable Activity Trackers That Motivate Women to Increase Physical Activity: Mixed Methods Study. JMIR Form Res. 2023;7:e48704. doi: 10.2196/48704.

75. Phipps J, Passage B, Sel K, et al. Early adverse physiological event detection using commercial wearables: challenges and opportunities. NPJ Digit Med. 2024;7(1):136. doi: 10.1038/s41746-024-01129-1.

76. Ryan JM, Navaneethan S, Damaso N, et al. Information theory reveals physiological manifestations of COVID-19 that correlate with symptom density of illness. Front Netw Physiol. 2024;4:1211413. doi: 10.3389/fnetp.2024.1211413. eCollection 2024.

77. Sharifi-Heris Z, Fortier MA, Rahmani AM, Sharifiheris H, Bender M. Feasibility of continuous smart health monitoring in pregnant population: A mixed-method approach. PLOS Digit Health. 2024;3(6):e0000517. doi: 10.1371/journal.pdig.0000517. eCollection 2024 Jun.

78. Liew SJ, Soon CS, Chooi YC, Tint MT, Eriksson JG. A holistic approach to preventing type 2 diabetes in Asian women with a history of gestational diabetes mellitus: a feasibility study and pilot randomized controlled trial. Front Clin Diabetes Healthc. 2023;4:1251411. doi: 10.3389/fcdhc.2023.1251411. eCollection 2023.

79. Kristiansson E, Fridolfsson J, Arvidsson D, et al. Validation of Oura ring energy expenditure and steps in laboratory and free-living. BMC Med Res Methodol. 2023;23(1):50. doi: 10.1186/s12874-023-01868-x.

80. Björkman K, Valkama M, Bruun E, et al. Heart Rate and Heart Rate Variability in Healthy Preterm-Born Young Adults and Association with Vitamin D: A Wearable Device Assessment. J Clin Med. 2023;12(24):7504. doi: 10.3390/jcm12247504.

81. Balsam D, Bounds DT, Rahmai AM, Nyamathi A. Evaluating the Impact of an App-Delivered Mindfulness Meditation Program to Reduce Stress and Anxiety During Pregnancy: Pilot Longitudinal Study. JMIR Pediatr Parent. 2023 Dec 25:6:e53933. doi: 10.2196/53933.

82. Bruce LK, Kasl P, Soltani S, et al. Variability of temperature measurements recorded by a wearable device by biological sex. Biol Sex Differ. 2023;14(1):76. doi: 10.1186/s13293-023-00558-z.

83. Croghan IT, Hurt RT, Fokken SC, et al. Stress Resilience Program for Health Care Professionals During a Pandemic: A Pilot Program. Workplace Health Saf. 2023;71(4):173-180. doi: 10.1177/21650799221093775

84. Erickson EN, Gotlieb N, Pereira LM, et al. Predicting labor onset relative to the estimated date of delivery using smart ring physiological data. NPJ Digit Med. 2023;6(1):153. doi: 10.1038/s41746-023-00902-y.

85. Jafarlou S, Lai J, Azimi I, et al. Objective Prediction of Next-Day's Affect Using Multimodal Physiological and Behavioral Data: Algorithm Development and Validation Study. JMIR Form Res. 2023 Mar 15;7:e39425. doi: 10.2196/39425.

86. Nolasco HR, Vargo A, Bohley N, Brinkhaus C, Kise K. Examining Participant Adherence with Wearables in an In-the-Wild Setting. Sensors (Basel). 2023;23(14):6479. doi: 10.3390/s23146479.

87. Padmanabha A, Choudhary S, Majidi C, Erickson Z. A multimodal sensing ring for quantification of scratch intensity. Commun Med (Lond). 2023;3(1):115. doi: 10.1038/s43856-023-00345-2.

88. Sanai F, Sahid AS, Huvanandana J, et al. Evaluation of a Continuous Blood Glucose Monitor: A Novel and Non-Invasive Wearable Using Bioimpedance Technology. J Diabetes Sci Technol. 2023;17(2):336-344. doi: 10.1177/19322968211054110.

89. Shiba SK, Temple CA, Krasnoff J, et al. Assessing Adherence to Multi-Modal Oura Ring Wearables From COVID-19 Detection Among Healthcare Workers. Cureus. 2023;15(9):e45362. doi: 10.7759/cureus.45362. eCollection 2023 Sep.

90. Mason AE, Hecht FM, Davis SK, et al. Detection of COVID-19 using multimodal data from a wearable device: results from the first TemPredict Study. Scientific reports. 2022;12(1):3463. doi: 10.1038/s41598-022-07314-0.

91. Mason AE, Kasl P, Hartogensis W, et al. Metrics from Wearable Devices as Candidate Predictors of Antibody Response Following Vaccination against COVID-19: Data from the Second TemPredict Study. Vaccines (Basel). 2022;10(2):264. doi: 10.3390/vaccines10020264.

92. Alzueta E, de Zambotti M, Javitz H, et al. Tracking Sleep, Temperature, Heart Rate, and Daily Symptoms Across the Menstrual Cycle with the Oura Ring in Healthy Women. Int J Womens Health. 2022:14:491-503. doi: 10.2147/IJWH.S341917. eCollection 2022.

93. Cao R, Azimi I, Sarhaddi F, et al. Accuracy Assessment of Oura Ring Nocturnal Heart Rate and Heart Rate Variability in Comparison With Electrocardiography in Time and Frequency Domains: Comprehensive Analysis. J Med Internet Res. 2022;24(1):e27487. doi: 10.2196/27487.

94. Conroy B, Silva I, Mehraei G, et al. Real-time infection prediction with wearable physiological monitoring and AI to aid military workforce readiness during COVID-19. Sci Rep. 2022;12(1):3797. doi: 10.1038/s41598-022-07764-6.

95. de Vries H, Kamphuis W, van der Schans C, Sanderman R, Oldenhuis H. Trends in Daily Heart Rate Variability Fluctuations Are Associated with Longitudinal Changes in Stress and Somatisation in Police Officers. Healthcare (Basel). 2022;10(1):144. doi: 10.3390/healthcare10010144.

96. Fonseka RD, Natarajan P, Maharaj MM, Rooke K, Mobbs RJ. Two-year continuous data capture using a wearable sensor to remotely monitor the surgical spine patient: a case report. J Spine Surg. 2022;8(1):170-179. doi: 10.21037/jss-21-89.

97. Jimah T, Kehoe P, Borg H, et al. A Micro-Level Analysis of Physiological Responses to COVID-19: Continuous Monitoring of Pregnant Women in California. Front Public Health. 2022:10:808763. doi: 10.3389/fpubh.2022.808763. eCollection 2022.

98. Miller DJ, Sargent C, Roach GD. A Validation of Six Wearable Devices for Estimating Sleep, Heart Rate and Heart Rate Variability in Healthy Adults. Sensors (Basel). 2022;22(16):6317. doi: 10.3390/s22166317.

99. Niela-Vilen H, Azimi I, Suorsa K, et al. Comparison of Oura Smart Ring Against ActiGraph Accelerometer for Measurement of Physical Activity and Sedentary Time in a Free-Living Context. Comput Inform Nurs. 2022;40(12):856-862. doi: 10.1097/CIN.0000000000000885.

100. Rovini E, Galperti G, Manera V, et al. A wearable ring-shaped inertial system to identify action planning impairments during reach-to-grasp sequences: a pilot study. J Neuroeng Rehabil. 2021;18(1):118. doi: 10.1186/s12984-021-00913-4.

101. Ahn C, Lee S, Lee J, et al. Impact of a Smart-Ring-Based Feedback System on the Quality of Chest Compressions in Adult Cardiac Arrest: a Randomized Preliminary Study. Int J Environ Res Public Health. 2021;18(10):5408. doi: 10.3390/ijerph18105408.

102. Henriksen A, Johannessen E, Hartvigsen G, Grimsgaard S, Hopstock LA. Consumer-Based Activity Trackers as a Tool for Physical Activity Monitoring in Epidemiological Studies During the COVID-19 Pandemic: Development and Usability Study. JMIR Public Health Surveill. 2021;7(4):e23806. doi: 10.2196/23806.

103. Jimah T, Borg H, Kehoe P, et al. A Technology-Based Pregnancy Health and Wellness Intervention (Two Happy Hearts): Case Study. JMIR Form Res. 2021;5(11):e30991. doi: 10.2196/30991.

104. Lee S, Song Y, Lee J, et al. Development of Smart-Ring-Based Chest Compression Depth Feedback Device for High Quality Chest Compressions: A Proof-of-Concept Study. Biosensors (Basel). 2021;11(2):35. doi: 10.3390/bios11020035.

105. Moshe I, Terhorst Y, Opoku Asare K, et al. Predicting Symptoms of Depression and Anxiety Using Smartphone and Wearable Data. Front Psychiatry. 2021:12:625247. doi: 10.3389/fpsyt.2021.625247. eCollection 2021.

106. Kinnunen H, Rantanen A, Kenttä T, Koskimäki H. Feasible assessment of recovery and cardiovascular health: accuracy of nocturnal HR and HRV assessed via ring PPG in comparison to medical grade ECG. Physiol Meas. 2020;41(4):04NT01. doi: 10.1088/1361-6579/ab840a.

107. Maijala A, Kinnunen H, Koskimäki H, et al. Nocturnal finger skin temperature in menstrual cycle tracking: ambulatory pilot study using a wearable Oura ring. BMC Womens Health. 2019;19(1):150. doi: 10.1186/s12905-019-0844-9.
